# Structural Sensitivity without Chirality: Observation of Magnetic Raman Optical Activity outside Resonance

**DOI:** 10.1021/jacs.5c22470

**Published:** 2026-03-04

**Authors:** Moumita Das, Petr Bouř

**Affiliations:** † Institute of Organic Chemistry and Biochemistry, Academy of Sciences, Flemingovo náměstí 2, 16610 Prague, Czech Republic; ‡ Department of Analytical Chemistry, 534467 University of Chemistry and Technology, Technická 5, 16628 Prague, Czech Republic

## Abstract

Magnetic Raman optical activity (MROA) has been considered to be confined to a few systems fulfilling the resonance conditions. In a far-from-resonance (FFR) case when the systems do not absorb the excitation radiation, it has not been reported. However, we find it present in many common organic molecules. The underlying theory was elaborated, and a procedure for quantum chemical simulations of MROA intensities was implemented. The spectral features predicted at the density functional theory (DFT) level reasonably agree with the observations, describe the trends in the experimental data, and allow one to understand the phenomenon more deeply. It appears that not only do molecules have specific MROA patterns but the intensities are also very much dependent on the conformation. The sensitivity to the structure in solutions without the need for intrinsic molecular chirality makes MROA a unique tool for analytical chemistry and potentially usable for conformational studies of both chiral and achiral molecules in solutions and other liquids.

## Graphical abstract

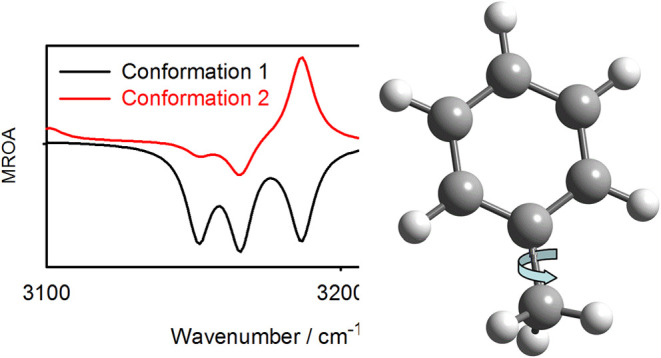

## Introduction

Contemporary chemistry is thirsty for means to monitor the molecular structure in solutions and liquids. Therefore, Raman optical activity (ROA) increasingly attracts attention as it provides extremely efficient means to study molecular structures and properties.^1,2^ From its discovery in 1973,^3^ ROA spectroscopy developed into many branches. The most common form is so-called far-from-resonance (FFR) ROA, when the sample, solution, or pure liquid does not absorb the incident laser radiation. It has been applied in many remarkable cases, such as the determination of the absolute configuration of isotopically chiral neopentane^4^ or the showcase molecule bromochlorofluoromethane,^5^ and has become a standard tool in peptide, protein, sugar, and nucleic acid conformational studies.^6–9^ In the case of methyloxirane, the spectrum of its gas phase has been reported as well.^10^ The performance of the method led to several commercially available ROA spectrometers that can be bought today, such as those from BioTools based on ref 11, Zebr,^12^ and Enantios.^13^

More recently, the so-called preresonance or resonance ROA (RROA) has been pursued vigorously as well, as it promises a stronger signal and a deeper view into vibrational and electronic molecular properties.^14–16^ In this case, the frequency of the excitation laser radiation is close to that of an electronic transition in the molecule. In the presence of the magnetic field, RROA provided information on the ground and excited vibrational states of dia- and paramagnetic gases.^17,18^ For carotenoid dyes, an interesting phenomenon, aggregation-induced ROA (AIROA), could be used to distinguish various aggregate types.^19^ In a broader sense, this category also comprises surface-enhanced ROA (SEROA) relying on plasmon resonance.^20,21^ However, resonance experiments have brought about many drawbacks and challenges. For example, only since 2020, it has been possible to measure solution RROA accurately, separating it from the ECD-Raman effect,^22^ which by itself developed into a self-standing spectroscopic method.^23^ Another problem is the instability of some systems under the resonance.

Apart from ROA, typical techniques that can be used to study molecular structures in solution include electronic^24^ and vibrational circular dichroism,^25^ nuclear magnetic resonance,^26^ or, to some extent, cryo-electron microscopy.^27^ The chiroptical methods are in general limited by the chirality that a molecule has to have to produce any signal. This restriction can, in principle, be removed when the system is placed in a magnetic field. For example, magnetic electronic circular dichroism (MCD) became a viable alternative to natural electronic circular dichroism for a range of molecules, such as porphyrins or fullerenes.^28–30^ Nevertheless, MCD is restrained to systems possessing suitable chromophores, ideally absorbing in the visible range. Magnetic vibrational circular dichroism (MVCD) in principle does not need such chromophores; however, the measurements are difficult and ideally require superconductor magnets as the effect is weak, especially for low-symmetry molecules.^31,32^ Therefore, magnetic ROA (MROA) may be a better alternative.

Previous MROA reports mostly focused on strongly resonant (absorbing) high-spin and high-symmetry systems, such as IrCl_6_^2–^, CuBr_4_^2–^ ^33,33^ and porphyrins.^34,35^ These included electronic Raman scattering^36^ and MROA of cytochrome *c*.^37^ FFR MROA is believed to be very weak. In addition, interpretation of the experiments was problematic. In spite of a general theory available,^35,37^ its implementation into reliable software allowing to connect the observed spectra to a concrete molecular structure has been missing.

In the present work, however, we report many FFR MROA observations, which were possible with our magnetic cell constructed originally for the measurement of diamagnetic resonance Raman optical activity^18^ and a sensitive dual-detector Zebr spectrometer.^12^ This kind of spectra could provide several advantages; unlike the electronic methods, no particular chromophore is needed; unlike Raman scattering, the magnetic structure-sensitive component is involved; and unlike natural ROA, MROA is applicable to nonchiral molecules.

In addition, semiclassical perturbation theory^35^ has been elaborated and implemented at common density functional theory (DFT) and time-dependent DFT (TDDFT)^38^ levels. Although amendable to future improvements, the computational apparatus reproduces the observed trends and seems to provide a solid basis for interpretation and understanding of the experiments.

## Results and Discussion

A common backscattering scattered circular polarization (SCP) modulation setup^1^ was used, where the sample is irradiated by an unpolarized 532 nm laser radiation and the difference between right- and left-circular polarized light intensities is detected (Figure 1). The sample is held in a magnetic field of about 1 T, parallel to the laser beam, realized by permanent neodymium magnets.^18^

**Figure 1:**
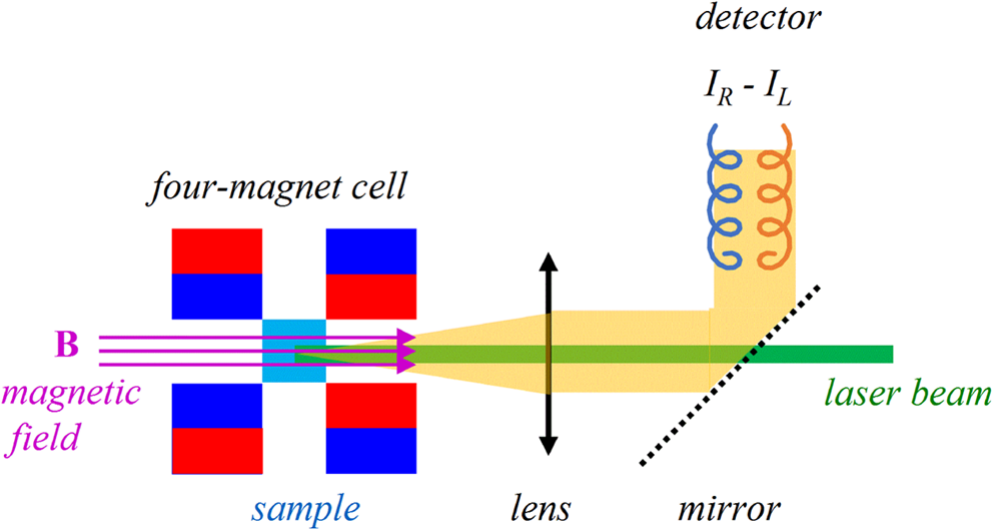
Schematic representation of the geometry of the scattered circular polarization (SCP) backscattering MROA experiment.

### MROA of Aromatics

In this arrangement, far-from-resonance MROA has been observed for a wide range of chemicals, albeit with different signal-to-noise ratios. Rich and high-quality spectra provide aromatic compounds; experimental MROA and Raman spectra of benzene, toluene, and pyridine are compared in the upper part of Figure 2. One can see that each molecule has a characteristic MROA pattern, similar to that for Raman, and the ability of MROA bands to be both positive and negative makes them even more useful for analytical chemistry. The normalized circular intensity difference (CID, MROA to Raman ratio, cf. ref 35) is rather low, typically 10^–5^–10^–4^; nonetheless, the spectra could be measured reliably, as was checked by the comparison of the signals under opposite magnet orientations [cf. Figure S1 for raw spectra; idealized experimental spectra, “(north – south)/2” for MROA and “(north + south)/2” for Raman, are shown in Figure 2]. In general, the strongest Raman bands also provide strong MROA, although individual signals differ. For example, Raman-strong CH stretching modes have weaker MROA in comparison to some aromatic ring vibrations. For all of the compounds, the main ring breathing mode displayed at the top of Figure 2 gives a strong negative MROA band.

**Figure 2:**
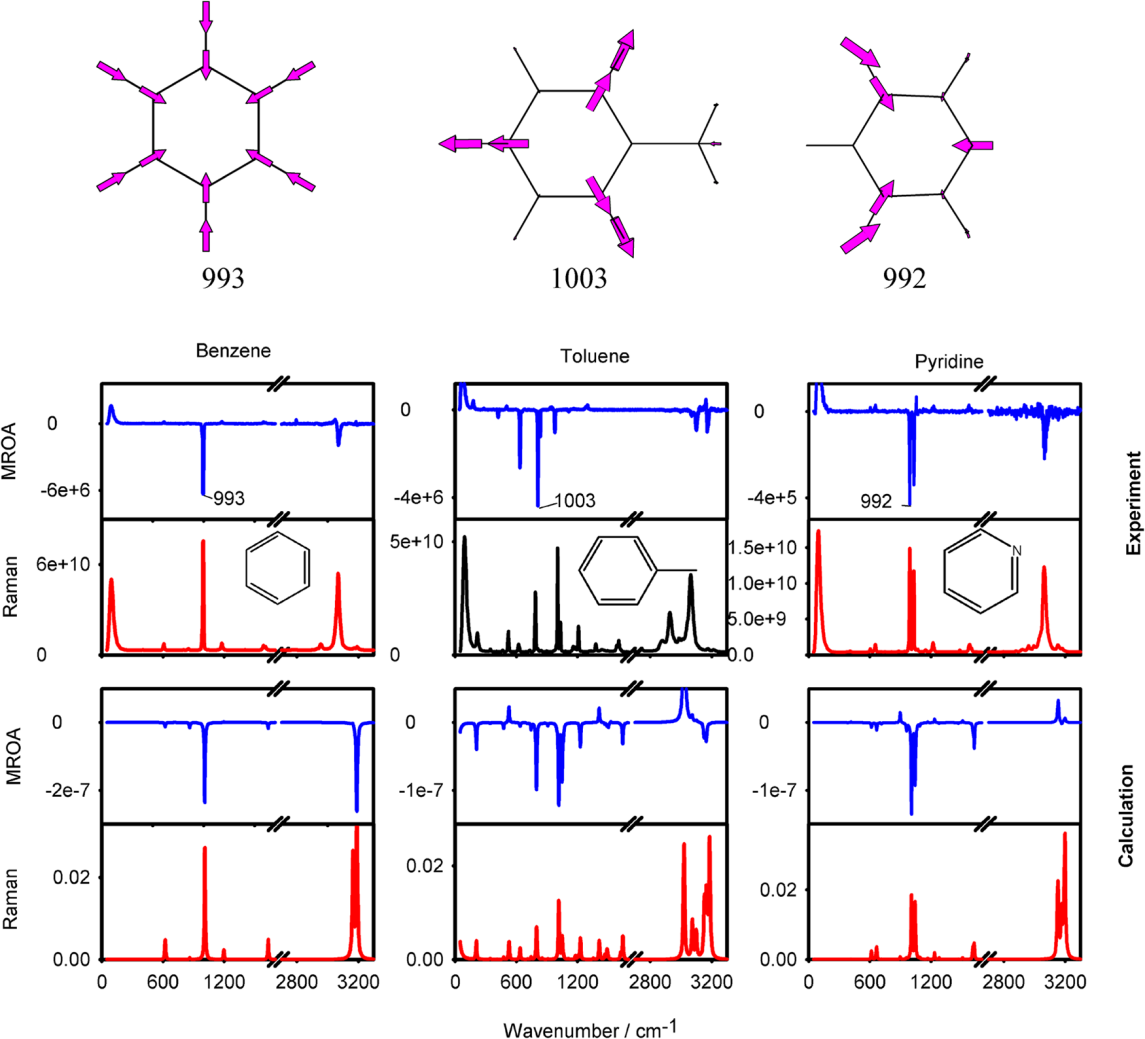
Experimental and calculated MROA and Raman spectra of the three aromatic compounds. Examples of vibrational modes providing the strongest signals are plotted at the top.

### Smaller Molecules and Ions

To explore the phenomenon for a broader class of chemicals, we focused on smaller molecules that can be measured at a high concentration to keep measurement times realistic. In addition, the experiment can be verified by spectral simulations in these cases. They include sodium acetate, tetrachloromethane, dimethyl sulfoxide (Figure 3), sulfuric acid, phosphoric acid, sodium thiosulfate, pyrrole, 1-methylpyrrole, and ethanol (Figure S2). The data indicate that MROA seems to be omnipresent, although some experiments remain challenging. For example, in Figure 3, for acetate, the CH_3_COO^–^ MROA signals occur on a baseline, most probably related to the aqueous environment. Neat liquids, such as CCl_4_ and DMSO, provide very strong and clean spectra.

**Figure 3:**
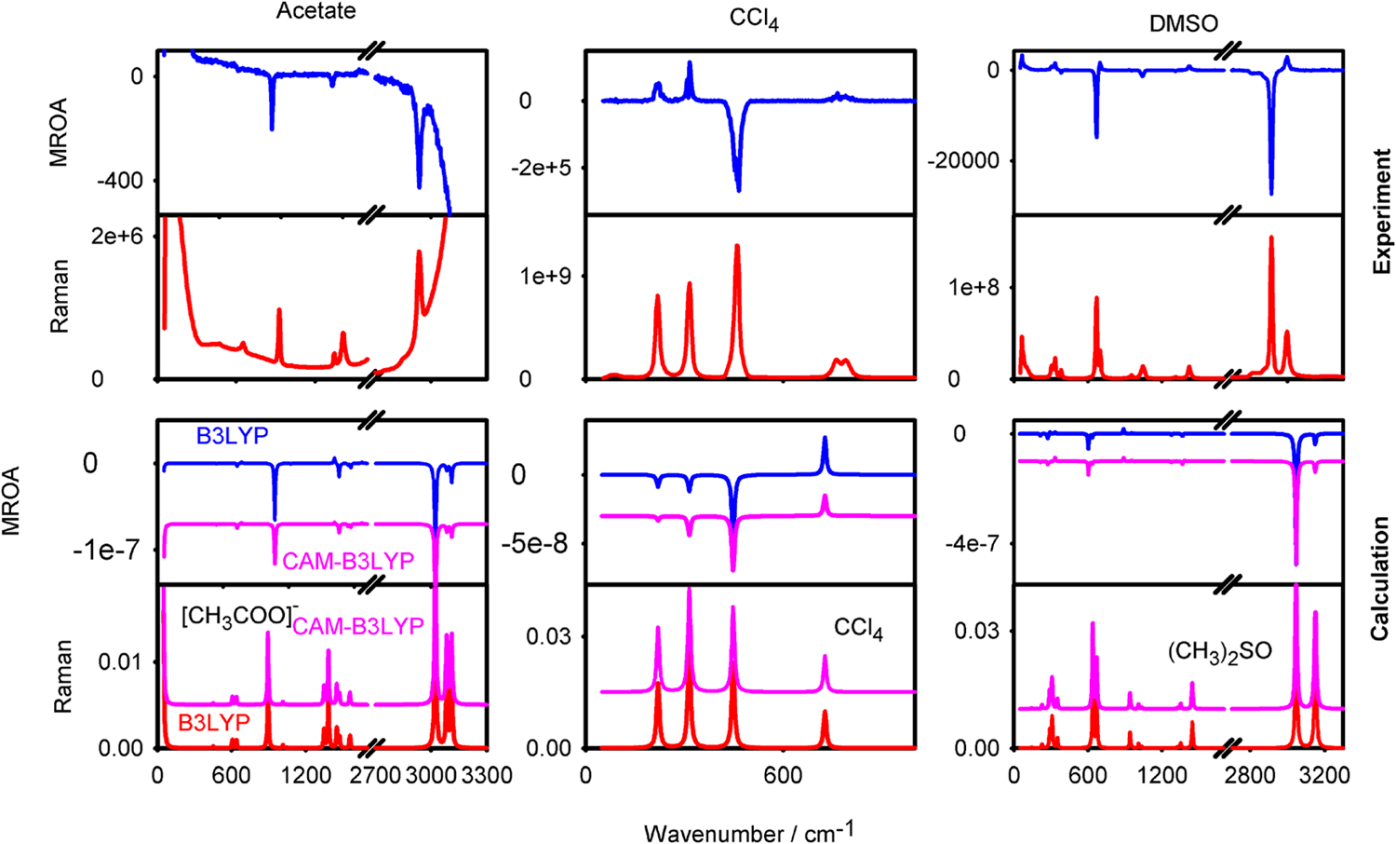
Experimental (top) and calculated (bottom) MROA and Raman spectra of sodium acetate, tetrachloromethane (CCl_4_), and dimethyl sulfoxide (DMSO).

For the theory, apart from the default B3LYP functional,^39^ its CAM-B3LYP variant with the Coulomb attenuation^40^ was used as well. Similarly, as for the larger aromatic compounds, the simulations reproduce the largest signals and trends in the relative intensity patterns well. The two functionals give nearly the same results, mostly differing in intensities of some small bands. In the case of 1-methylpyrrole, however, CAM-B3LYP flipped signs of small bands around 1500 cm^–1^, in favor of the comparison with the experiment (Figure S2).

Overall, the computed spectra at the lower part of Figure 3 reasonably well follow the experimental trends, although the theoretical limitations are apparent. Some weaker bands are predicted with the wrong signs. This is in line with DFT accuracy for other chiroptical properties, such as optical rotatory dispersion (ORD), where trends are in general well captured, while individual values are predicted with a relatively large error.^41^ The computed CIDs are mostly lower than the experimental ones (Table 1). It should be noted that the experimental values are very much affected by the instrumental noise and resolution and should be taken with caution as well. In particular, the narrowness of the peaks covering only a few pixels of the coupled-charge device detector makes precise intensity integration difficult. The low-frequency signal (below ∼200 cm^–1^) cannot be predicted with the monomolecular simulations, as it often stems from intermolecular interactions.^42^ In spite of these limitations, the computations do provide the most prominent spectral features, capture well the differences between different chemicals, and provide a good basis for understanding, prediction, and interpretation of the phenomenon.

**Table 1:** Calculated and Experimental Frequencies (ω, in cm^–1^) and MROA/Raman Ratios (CID) for Selected Vibrational Bands in the Aromatic Compounds

	ω_exp_	ω_cal_	CID_exp_ × 10^4^	CID_cal_ × 10^4^
benzene	994	1011	–0.58	–0.13
	3068	3164	–0.15	–0.01
toluene	786	797	–0.88	–0.35
	1003	1015	–0.85	–0.11
	1210	1227	–0.21	–0.13
pyridine	992	3165	–0.18	–0.11
pyrrole	1138	1168	–2.23	–0.23
	1382	1411	–0.70	–0.05
	1470	1493	–1.09	–0.13
	3135	3260	–1.88	–0.02

### Conformational Dependence of the Spectra

We find the sensitivity of MROA to the conformation interesting and useful as it, unlike natural optical activity, could potentially be used for conformational studies of nonchiral molecules. Two such examples are shown in Figure 4. Sulfuric acid is rather an academic case only since the experimental spectra are very noisy and the modeling must currently be limited to single molecules, ignoring the environment and complicated dissociation equilibria in the actual sample. Nevertheless, we are not aware of other analytical methods that could be used for studies of the H_2_SO_4_ geometry in the liquid phase. Yet, MROA bands radically differ for the different conformers. This contrasts with the differences in Raman bands, mostly restricted to frequency shifts.

**Figure 4:**
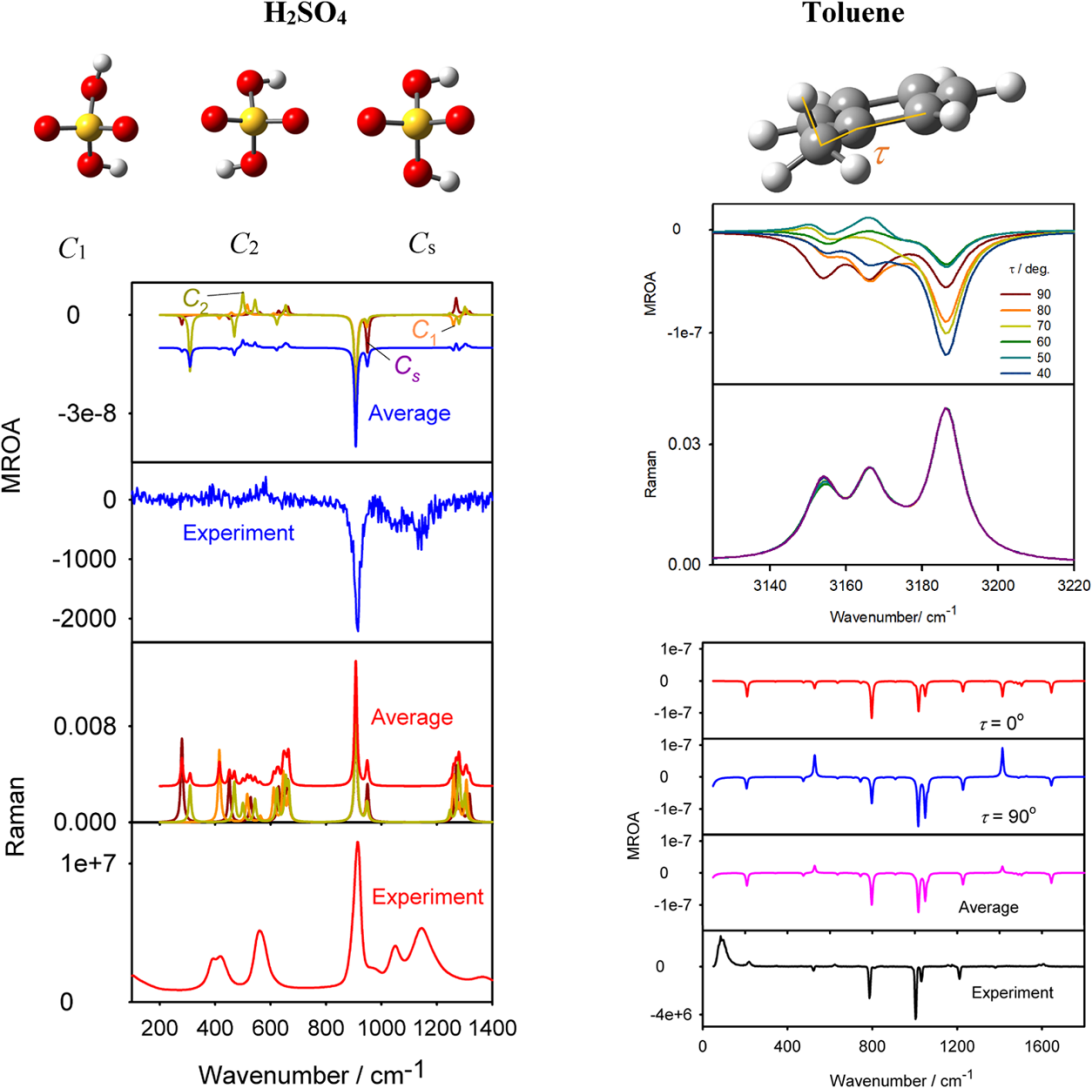
MROA and Raman spectra simulated (B3LYP/6-311++G**/PCM) for sulfuric acid and toluene conformers and the experiments. For the acid, the calculated wavenumber scale was shifted to match the largest experimental signal, for easier comparison. For toluene, spectra in the CH stretching region calculated for different torsion angles are magnified at the top.

For toluene, one can see that vibrational modes centered at the methyl group are affected most, such as C–H stretching calculated at around 3150 cm^–1^, CH_3_ umbrella CH bending (1411 cm^–1^), and benzene ring deformation (523 cm^–1^). The MROA conformational dependence contrasts with the near-conformational indifference of the parent Raman signal. A comparison of the CH stretching part to the experiment is problematic because of large anharmonicities in these vibrations;^43^ however, in the fingerprint region, averaging of the two conformers does seem to bring the theoretical MROA pattern closer to the measured one. This is consistent with the low energy barrier of the methyl rotation in this molecule.^44^

Two other examples exhibit a similar behavior. Four conformers of phosphoric acid, similar to those of H_2_SO_4_, give distinct MROA spectra, and the averaging leads to a better agreement with the experiment (Figure S3). As a “biomolecular” application, we also measured the MROA of diglycine (glycil-glycine zwitterion) and simulated the spectra for the *cis*- and *trans*-conformer (Figure S4). Again, their spectra are quite different, and the *trans*-conformer gives a better match with the experiment, as expected from conformer relative energies.^45^ A more quantitative assessment is complicated by the experimental noise since only a relatively low concentration for diglycine could be achieved.

### Terahertz MROA

Some compound spectra, for example, those shown in Figure 2, exhibit a relatively strong MROA in the lowest-wavenumber “terahertz” region, approximately below 200 cm^–1^. The vibrations therein can be mostly assigned to intermolecular motions.^42^ Thus, the spectroscopy can potentially be used to study these molecular motions, intermolecular interactions, and the structure of the liquids. Since the organic molecules are currently too big to be modeled with our computational means, we attempted to verify these results with water. Indeed, as follows from Figure 5, the intermolecular and monomolecular rotational and translational modes do support a strong MROA. The resultant spectrum reproduces the experiment, where, for example, the lower-frequency (∼<1000 cm^–1^) signal of the opposite sign is the highest one (>∼3000 cm^–1^). However, note that the experimental signal of water is too weak for a more quantitative comparison. In addition, the OH stretching region is prone to instrumental artifacts due to the strong parent Raman signal.

**Figure 5:**
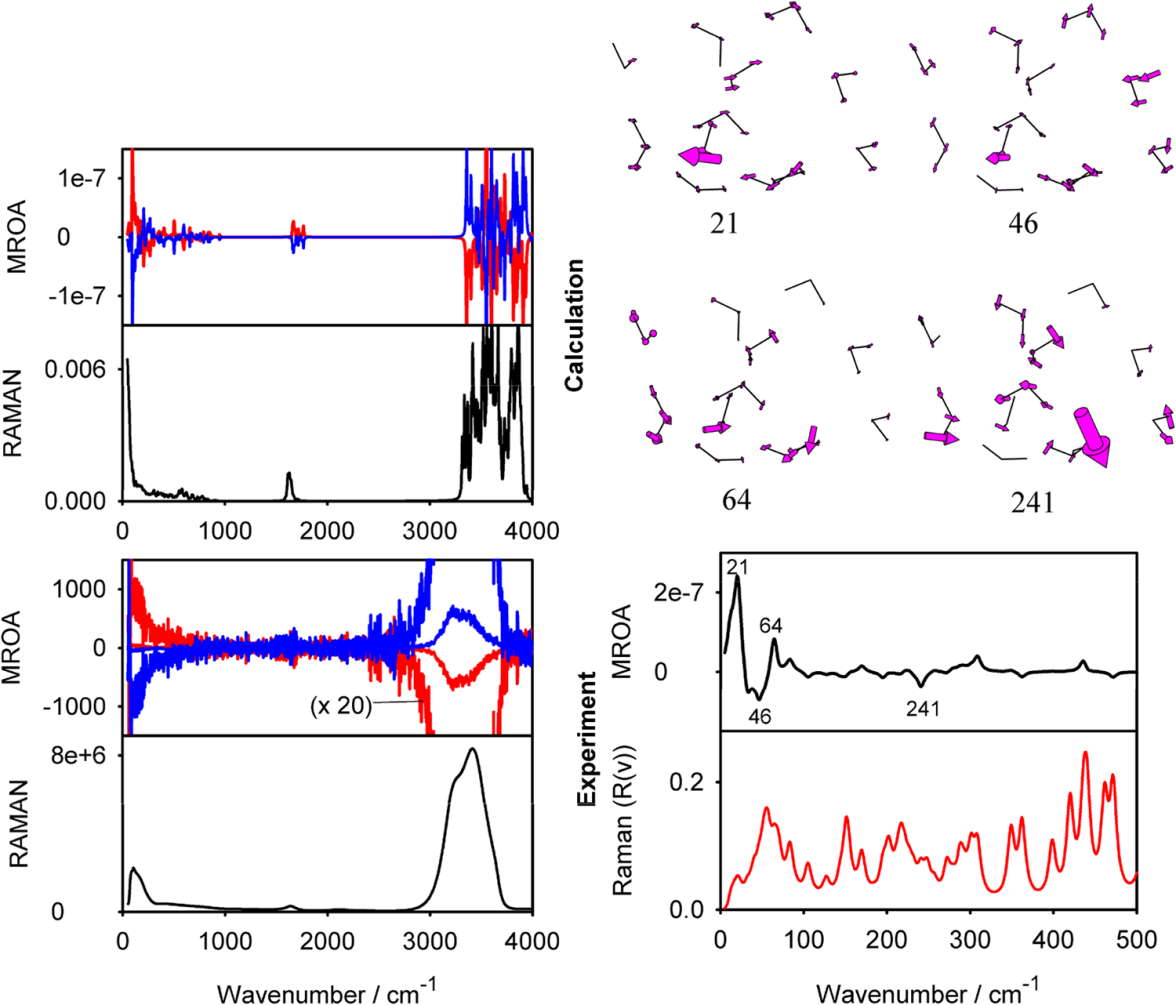
(Left) Simulated and experimental MROA and Raman spectra of water for two magnet orientations (red/blue). The simulation is an average from 10 water clusters obtained from random MD snapshots. (Right) Simulated MROA and Raman spectra for one cluster below 500 cm^–1^. Selected vibrational normal modes are displayed above. In this case, Raman intensities are given in the “*R*(*v*)” representation,^46^ *R*(*v*) = *v*[1 – exp­(−*hv*/*kT*)]*I*(*v*), which is convenient for the lowest-wavenumber region.

### Deeper Insight from the Theory

As detailed in the Experimental Section and the Supporting Information (SI), MROA intensities are dependent on changes of molecular transition polarizabilities caused by the magnetic field. These are collected in a tensor β describing the dependence of molecular polarizability on the geometry and magnetic field. The theory seems to reproduce the experimental observations quite well; nevertheless, one has to understand its limits. The sum-over-states (SOS) methodology used to obtain β has been successful in the past, for example, for computation of MCD intensities.^29,47,48^ In principle, all electronically excited molecular states from TDDFT are needed. This is not possible for large molecules. Fortunately, as exemplified for benzene in Figure 6, the convergence of MROA intensities is reasonable; 200–500 states already give a good estimate of the limit result. Another drawback of the SOS formulation is a potential origin dependence of the results, which, in the present study, was avoided by placing the origin in the mass center (cf. SI).

**Figure 6:**
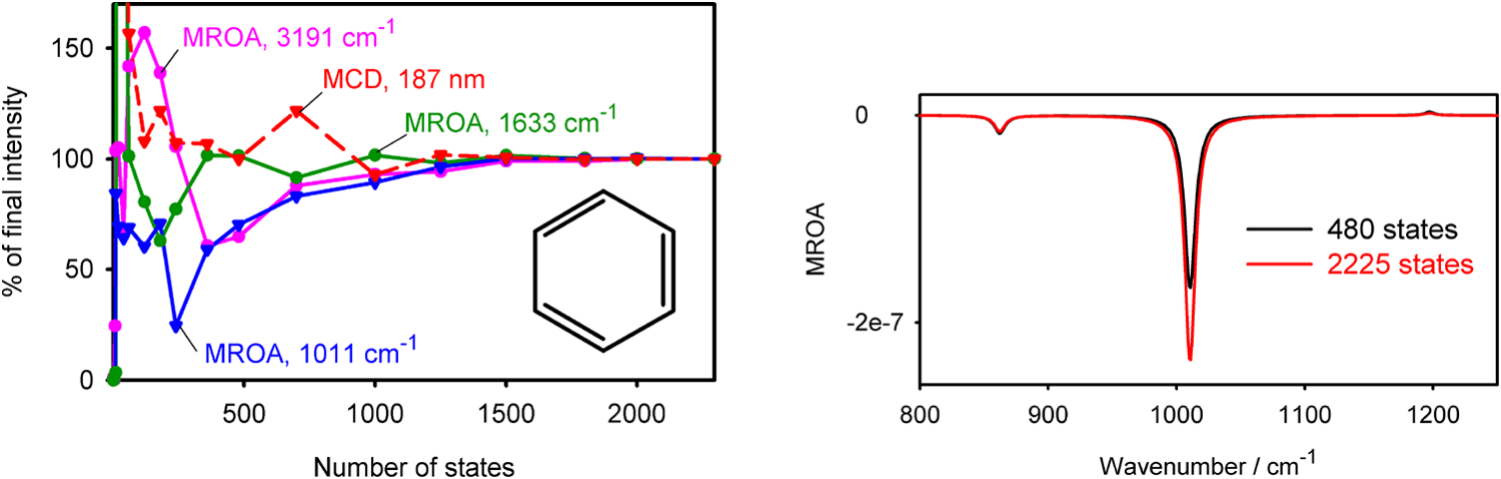
(Left) Benzene; convergence of MROA intensities of selected vibrational transitions and the 187 nm MCD band. (Right) Part of the MROA spectrum; a B3LYP/6-311++G** simulation.

The SOS computations involve a full Hamiltonian diagonalization with the field included in a nonperturbative way. Therefore, nonlinear effects and degenerations in electronic energies are consistently included. However, for laboratory conditions, MROA intensities depend linearly on the field. This is shown for a toluene band in Figure 7a. Only for very high unrealistic fields (>∼1000 T), visible deviations would occur. The linearity is a consequence of the small magnetic perturbation as compared to the differences of molecular electronic energies. From the experimental point of view, it would be obviously desirable to increase the field strength, to reduce the noise. This may be possible by incorporating superconductor magnets in future spectrometers.

**Figure 7:**
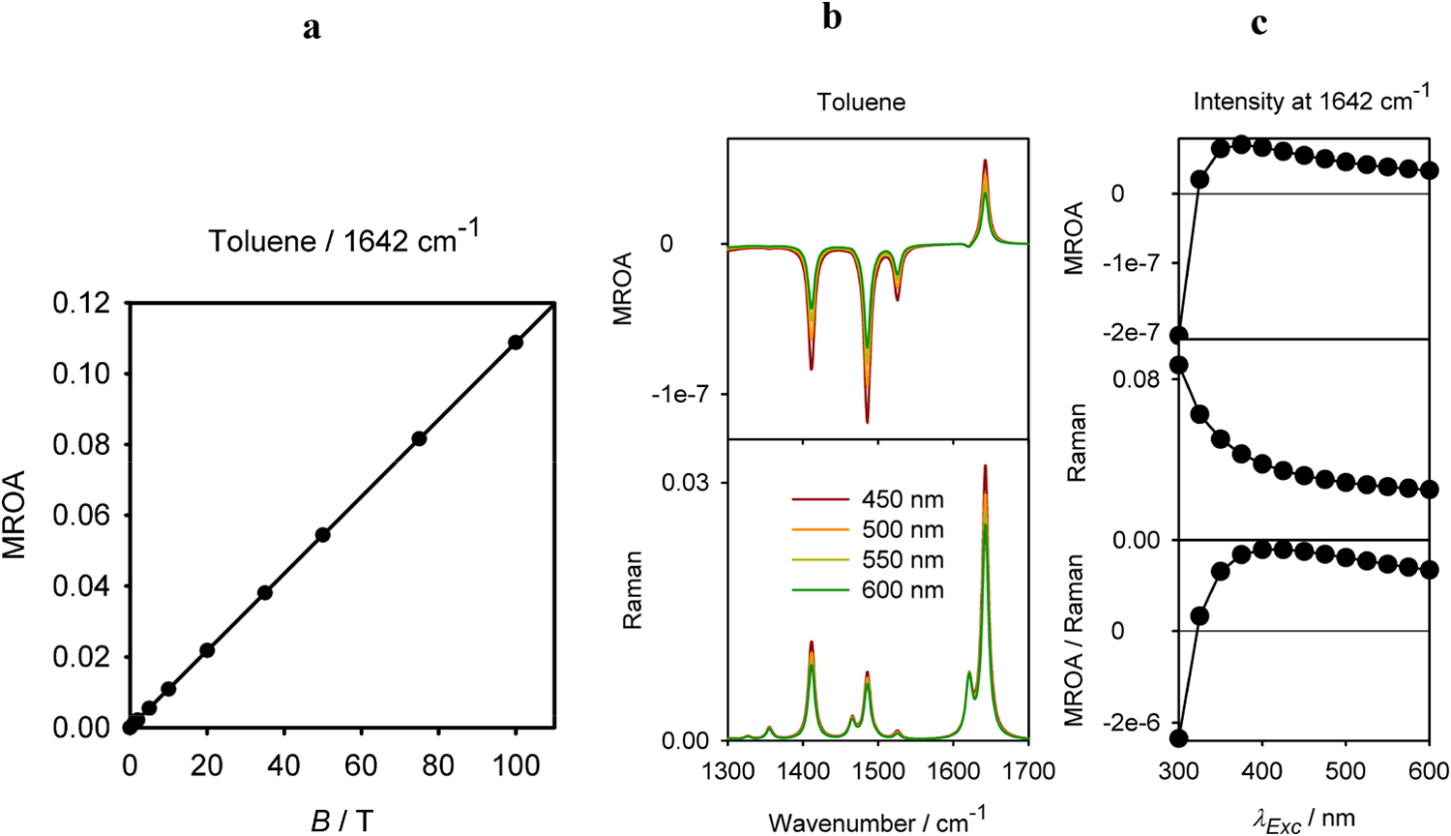
Toluene; (a) simulated dependence of the 1624 cm^–1^ MROA band intensity on the magnetic field, (b) MROA and Raman spectra simulated for four excitation wavelengths, and (c) MROA intensity of the 1624 cm^–1^ band dependent on the excitation wavelength.

Another property that is currently not possible to be studied experimentally is the MROA dependence on the excitation wavelength. Again, this is easy to investigate theoretically within the SOS method, as shown for toluene in Figure 7b,c. In the visible range (>400 nm), the spectral intensity only slowly increases toward shorter wavelengths, as does the MROA/Raman ratio (the so-called normalized circular intensity difference, CID).^35^ Only close to the toluene absorption threshold (∼275 nm)^49^ do the spectra change more radically. FFR MROA thus behaves as analogous properties dependent on tensor molecular responses to the magnetic field, such as ORD.^50^

The modeling thus provides justification and an understanding of the measured data. From the experimental point of view, it is important to realize that (1) MROA can be expected for any molecule, (2) polarizable compounds with π-electronic systems where energies of the electronic transitions are close to the laser excitation give the strongest signal, and (3) the differential signal comes from Raman scattering; thus, large Raman bands are in general accompanied by strong MROA. Within one molecule, the signal is not a plain sum of individual group contributions; a typical example is toluene, where we see signals both from the polarizable phenyl group as well as from the methyl group.

### Near-Resonance and Very-Far-from-Resonance Experiments

To further document the dependence of the spectra on the excitation wavelength–absorption threshold gap, we compare MROA and Raman spectra for two extreme cases. The results for ferro- and ferricyanide potassium salts documenting a near resonance are shown in Figure 8. Due to the limited concentration, the spectra were difficult to measure, and the experimental noise was quite large (cf. Figure S5 with the raw spectra). The ferricyanide exhibits a signal within ∼800 to 1000 cm^–1^, which is not simulated and which could be perhaps related to its partial decomposition or an electronic transition.^51^ Indeed, a nonlinearity was observed during the spectra accumulation (Figure S6). In spite of these problems, we see that ferricyanide MROA is much stronger, which corresponds to the narrower gap of this compound. The results also demonstrate the sensitivity of the MROA spectra to the oxidation number in these complexes. The structure and Raman spectra are very similar, whereas MROA of [Fe^III^(CN)_6_]^3–^ is much more intense than for [Fe^II^(CN)_6_]^4–^ and contains the extra sign information. Only the lowest vibrational band at 120 cm^–1^ gives similarly strong negative bands for both compounds.

**Figure 8:**
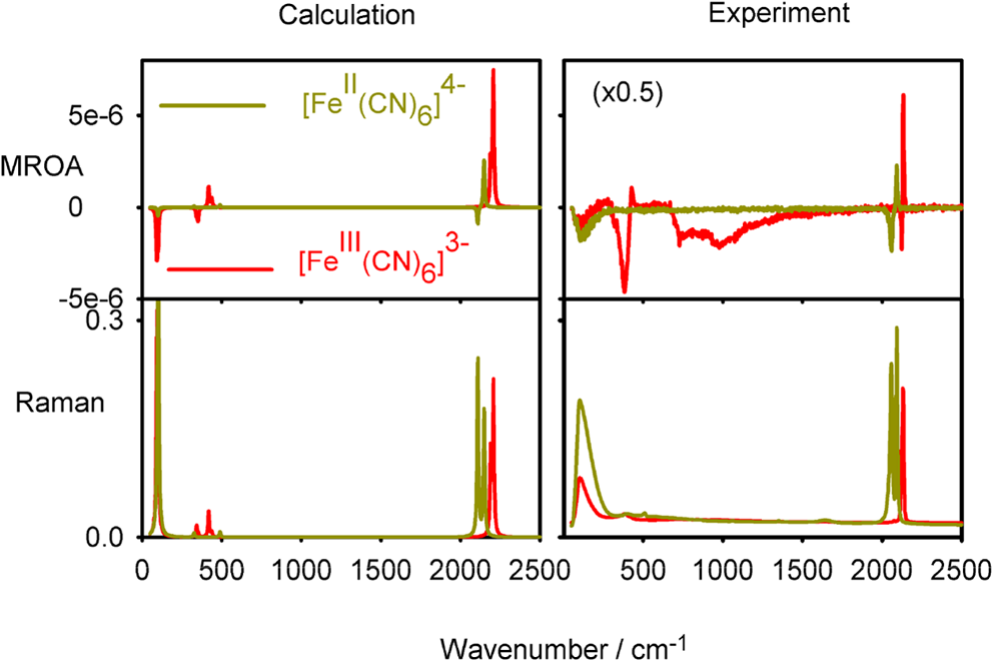
Calculated and experimental spectra of ferrocyanide and ferricyanide potassium salts.

An opposite extreme of a very large excitation wavelength–absorption threshold gap may be documented on α-pinene, where magnetic ROA is negligibly weak compared to (natural) ROA and hidden in noise. This behavior is nicely predicted by the theory (Figure S7), while the reliable simultaneous measurement of Raman, natural, and magnetic ROA spectra for this case remains an experimental challenge.

## Conclusions

The data show that far-from-resonance magnetic Raman optical activity is measurable for a wide range of molecules using a sensitive ROA spectrometer. To verify the experiments, we have also developed and implemented a theory compatible with routine time-dependent density functional computations usable for the spectral prediction and understanding. The results indicate that MROA intensities can be reasonably accurately calculated, which provides the necessary link between the spectral shapes and the molecular structure.

At the same time, many experimental and theoretical challenges remain. Currently, the measurement is difficult for solutions because of the weakness of the signal and the necessity of long accumulation times. Neither was MROA measurable for α-pinene, where it was obscured by natural ROA. The computations are dependent on lengthy numerical differentiation and somewhat hampered by the origin dependence. However, these obstacles may be removed in the future by the availability of more sensitive instruments and by analytical origin-independent theory implementations. Compared to its resonance branch, FFR MROA is more gentle to the samples. Combining the universalities of vibrational spectroscopy, not requiring special chromophores, magnetic properties, extremely sensitive to conformation, and achiral techniques, applicable regardless of molecular symmetry, the technique thus has clear potential in future chemical research.

## Experimental Section

### Raman and MROA Measurement

Commercial chemicals (Sigma-Aldrich) were used. The spectra were measured on a Zebr spectrometer^12^ at 20 °C, within 50–4550 cm^–1^, using a 532 nm excitation wavelength, the backscattering geometry, and the scattered circular polarization (SCP) modulation scheme. The samples were held in a 10 mm quartz cell from Hellma Analytics (600 μL) in a magnetic compartment^18^ producing a magnetic field of about 1 T. The laser power at the sample ranged from 60 to 250 mW and the data was collected for 7–22 h for different samples for each magnet orientation. Typical concentrations used for the solutions were around 1 M. Further experimental details are summarized in the SI.

### Theory of Magnetic ROA and Computations

We are using the semiclassical formulation^35^ where the excitation radiation polarizes the molecule, induces a time-dependent electric dipole and higher moments, which are then the sources of the detected radiation. Summarizing the details in the SI, for the backscattered SCP experiment, the sample is irradiated by unpolarized light, and the total scattered intensity is

1 $$I_{R}+I_{L}=\frac{K^{2}}{30}\left(\alpha_{\alpha \alpha }\alpha_{\beta \beta }^{*}+7\alpha_{\alpha \beta }\alpha_{\alpha \beta }^{*}\right)S_{0}$$

where *I*_R_/*I*_L_ are intensities of the right/left circularly polarized light, *K* is a constant, α is the transition (Raman) polarizability tensor, and *S*_0_ is the intensity of the excitation light. The Einstein summation convention is used throughout.

Under the presence of the magnetic field **B**, the polarizability becomes

2 $$\alpha_{\alpha \beta }=\alpha_{\alpha \beta }^{0}+\beta_{\alpha \beta ,\gamma }B_{\gamma }$$

where α^0^ is the polarizability at zero field, and β collects polarizability derivatives with respect to the field. The detected MROA intensity is then

3 IR−IL=215K2⁡Im⁡εαγδ(βαβ,γαδβ0*−ααβ0βγβ,δ*)S0Bz

where ε is the antisymmetric tensor.

The procedure used in this study for ab initio simulation of the spectra is summarized in Figure 9. The geometries were optimized by energy minimization at the B3LYP^39^/6-311++G** level using Gaussian software.^52^ The polarizable continuum model (PCM) was used to account for the environment. The CAM-B3LYP^40^ functional and a larger AUG-cc-pVTZ basis set on selected systems did not provide significantly different results.

**Figure 9:**
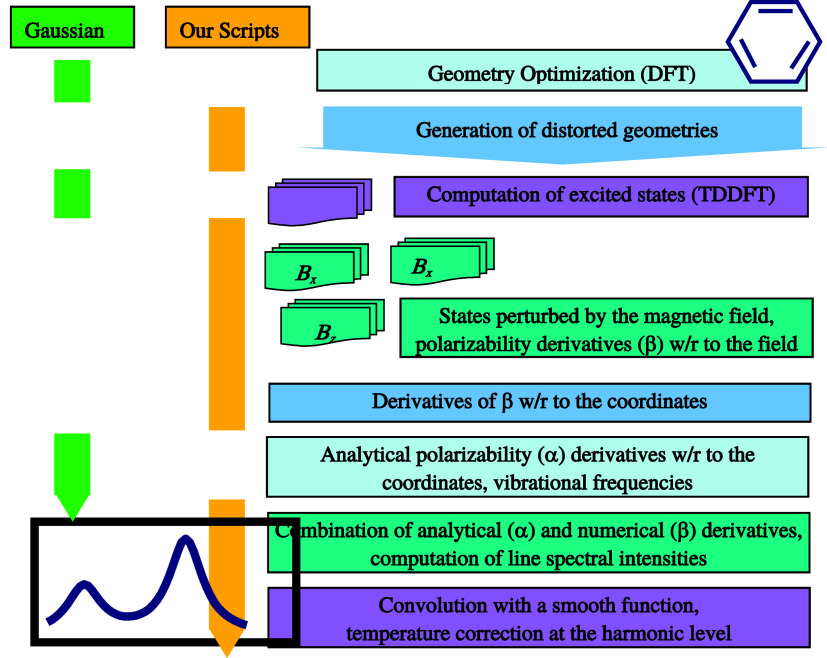
Main computation steps in the simulations of the MROA spectra.

Excited electronic states φ_*J*_ were obtained at the same level, using time-dependent DFT.^53^ These were used to diagonalize the Hamiltonian

4 $$H=H_{0}-m\cdot B$$

where *H*_0_ is its unperturbed part and **m** is the magnetic dipole moment operator. From the ground ( $\Psi_{0}\left(B\right)=\sum_{J}D_{0J}\phi_{J}$ ) and excited electronic states { $\Psi_{e}\left(B\right)=\sum_{J}D_{\mathrm{eJ}}\phi_{J}$ }, the electronic polarizability in the presence of the magnetic field was calculated as

5 $$\alpha_{E,\alpha \beta }\left(B_{\gamma }\right)=\frac{1}{\hbar }\sum_{e\neq 0}\left(\frac{\mu_{0e\alpha }\left(B_{\gamma }\right)\mu_{e0\beta }\left(B_{\gamma }\right)}{\omega_{e0}\left(B_{\gamma }\right)-\omega -i\Gamma }+\frac{\mu_{0e\beta }\left(B_{\gamma }\right)\mu_{e0\alpha }\left(B_{\gamma }\right)}{\omega_{e0}\left(B_{\gamma }\right)+\omega +i\Gamma }\right)$$

where Γ = 800 cm^–1^ accounts for the finite lifetime of the excited states,^54^ ω_*e*0_ is the angular frequency corresponding to the excitation energy, ω is the excitation frequency, *ℏ* is the reduced Planck constant, and the dipole moment matrix elements are

6 $$\mu_{0e\alpha }\left(B_{\gamma }\right)=\left\langle \Psi_{0}\left(B_{\gamma }\right)\right|\mu_{\alpha }\left|\Psi_{e}\left(B_{\gamma }\right)\right\rangle$$

This way, the desired polarizability derivatives could be calculated numerically as

7 $$\beta_{\alpha \beta ,\gamma }=\frac{\partial \alpha_{\alpha \beta }}{\partial B_{\gamma }}=\frac{\alpha_{\alpha \beta }\left(B_{\gamma }\right)-\alpha_{\alpha \beta }\left(0\right)}{B_{\gamma }}$$

In test computation, a double differentiation provided virtually the same results. The transition β-polarizabilities needed in eq 3 were calculated from numerical coordinate derivatives obtained as

8 $$\frac{\partial \alpha_{E,\alpha \beta }\left(B_{\gamma }\right)}{\partial R_{\varepsilon }}=\frac{\alpha_{E,\alpha \beta }\left(B_{\gamma },R_{\varepsilon }+\Delta \right)-\alpha_{E,\alpha \beta }\left(B_{\gamma },R_{\varepsilon }-\Delta \right)}{2\Delta }$$

where α_*E*,αβ_(*B*_γ_, *R*_ε_ + Δ) denotes the polarizability obtained for the geometry distorted by a Δ-shift of coordinate ε, Δ = 0.05 Å. Harmonic frequencies and transition α-derivatives were calculated by using Gaussian software. From the line intensities, smooth spectra were generated as

9 $$S\left(\nu \right)=\sum_{i}I_{i}{\left[1-\exp\left(-\frac{\hbar \omega_{i}}{\mathrm{kT}}\right)\right]}^{-1}{\left[4{\left(\frac{\omega -\omega_{i}}{\Delta }\right)}^{2}+1\right]}^{-1}$$

where *k* is the Boltzmann constant, the sum runs over vibrational transitions *i* with fundamental frequencies ω_*i*_, *T* is the temperature, and Δ = 10 cm^–1^.

## Supplementary Material



## Notes

Raw spectra, details about the measurement and theory, computational details, and test of the origin dependence of the results (PDF)
