# Supplementary material for: Structural Sensitivity without Chirality: Observation of Magnetic Raman Optical Activity outside Resonance
Source: J Am Chem Soc. 2026 Mar 4;148(10):11058–67. doi: 10.1021/jacs.5c22470 (PMC13003481; doi:10.1021/jacs.5c22470)
Supplement: Supplementary file 1 [file ja5c22470_si_001.pdf]

*Supporting Information*

# Structural Sensitivity without Chirality: Observation of Magnetic Raman Optical Activity outside Resonance

**Moumita Das[a,b] and Petr Bouř[a,b*]**

[a] Institute of Organic Chemistry and Biochemistry, Academy of Sciences, Flemingovo náměstí 2, 16610, Prague, Czech Republic

[b] Department of Analytical Chemistry, University of Chemistry and Technology, Technická 5, 16628 Prague, Czech Republic

[*] Corresponding author E-mail: bour@uochb.cas.cz

**Contents**

**Figure S1.** Raw Raman and MROA spectra of benzene, toluene, and pyridine.

**Figure S2.** Experimental and calculated MROA and Raman spectra of $H_2SO_4$, $H_3PO_4$, $Na_2S_2O_3$ (1 M water solution), pyrrole, 1-methylpyrrole and ethanol.

**Figure S3.** MROA a Raman spectra simulated for four conformers of phosphoric acid.

**Figure S4.** MROA a Raman spectra of Gly-Gly.

**Figure S5.** Raw Raman and MROA spectra of $K_3[Fe(CN)_6]$ and $K_4[Fe(CN)_6]$.

**Figure S6.** Examples of time-dependence of Raman and MROA spectra during the accumulation.

**Figure S7.** Calculated and experimental ROA and MROA spectra of α-pinene.

**Figure S8.** Experimental absorption spectra of measured systems.

**Figure S9.** Experimental MROA and Raman spectra of 1:1 pyridine : toluene mixture.

**Figure S10.** MROA and Raman spectra of sodium thiosulfate and acetate mixture.

**Figure S11.** MROA and Raman spectra of DMSO/glycerin mixtures.

**Figure S12.** Concentration-dependence of MROA and Raman spectra of sodium thiosulfate.

**Figure S13.** Raman scattering on a molecule.

**Figure S14.** Toluene MROA spectra calculated for two coordinate origins.

| | |
|---|---|
| **Spectra measurement** | S14 |
| **Theory of magnetic Raman optical activity** | S16 |
| **Computations of the derivatives of the polarizability** | S19 |
| **Origin dependence** | S21 |
| **Implementation** | S24 |

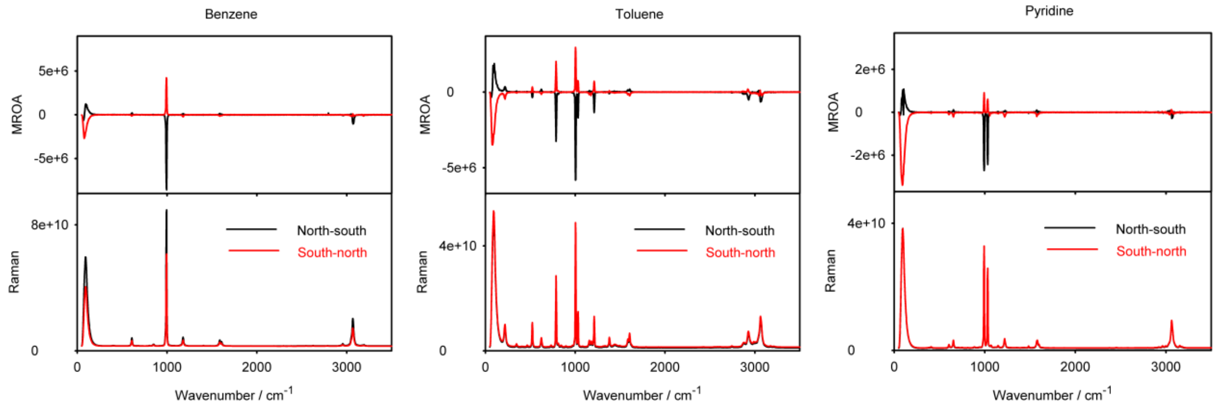


**Figure S1.** Raw Raman and MROA spectra of benzene, toluene, and pyridine. The experimental conditions are given below.

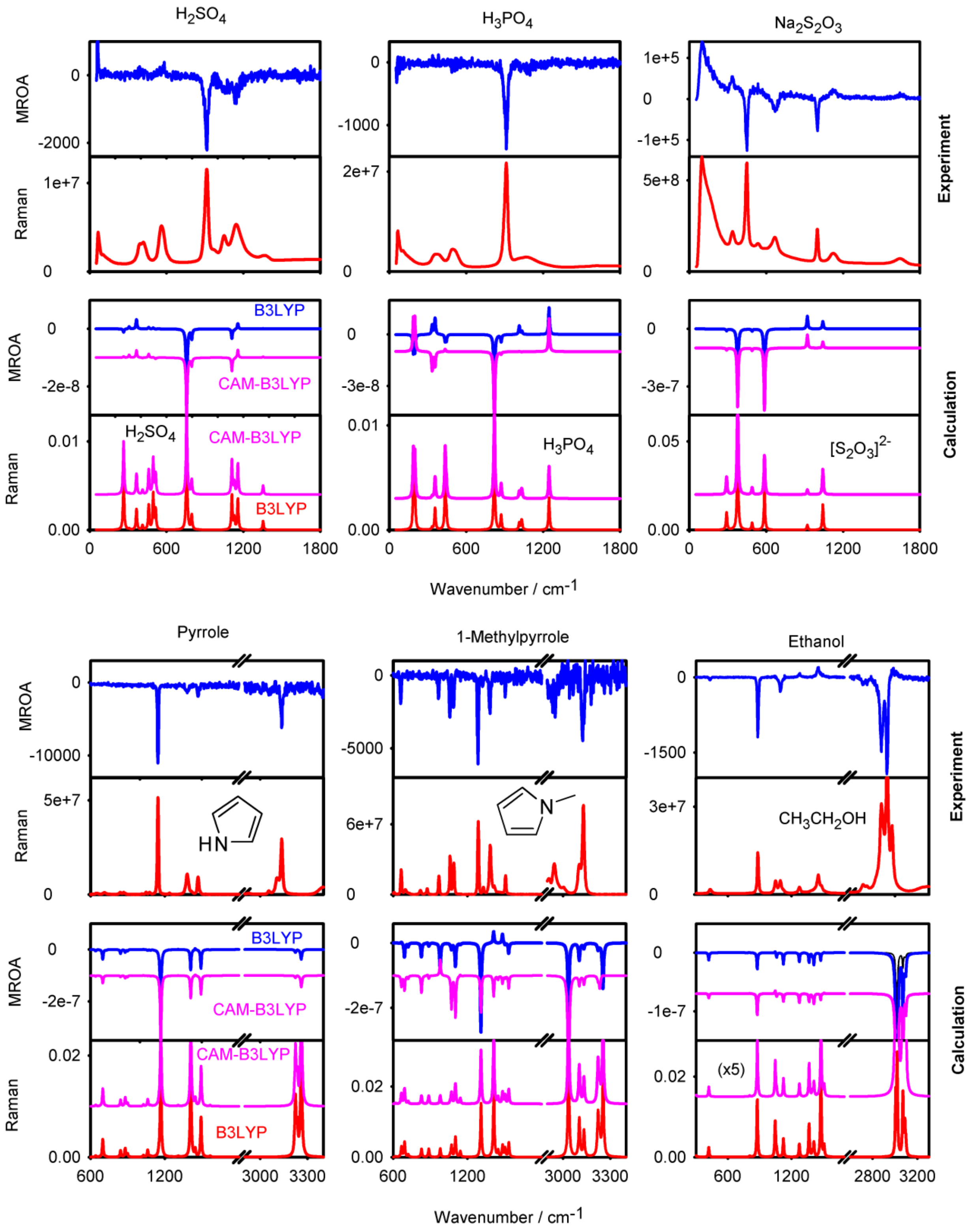


**Figure S2.** Experimental and calculated MROA and Raman spectra of $H_2SO_4$, $H_3PO_4$, $Na_2S_2O_3$ (1 M water solution), pyrrole, 1-methylpyrrole and ethanol. B3LYP and CAM-B3LYP computations are shown, performed with the 6-311++G** basis set and PCM solvent model. For $H_2SO_4$ and $H_3PO_4$, presence of dissociated forms in the sample was ignored in the simulations.

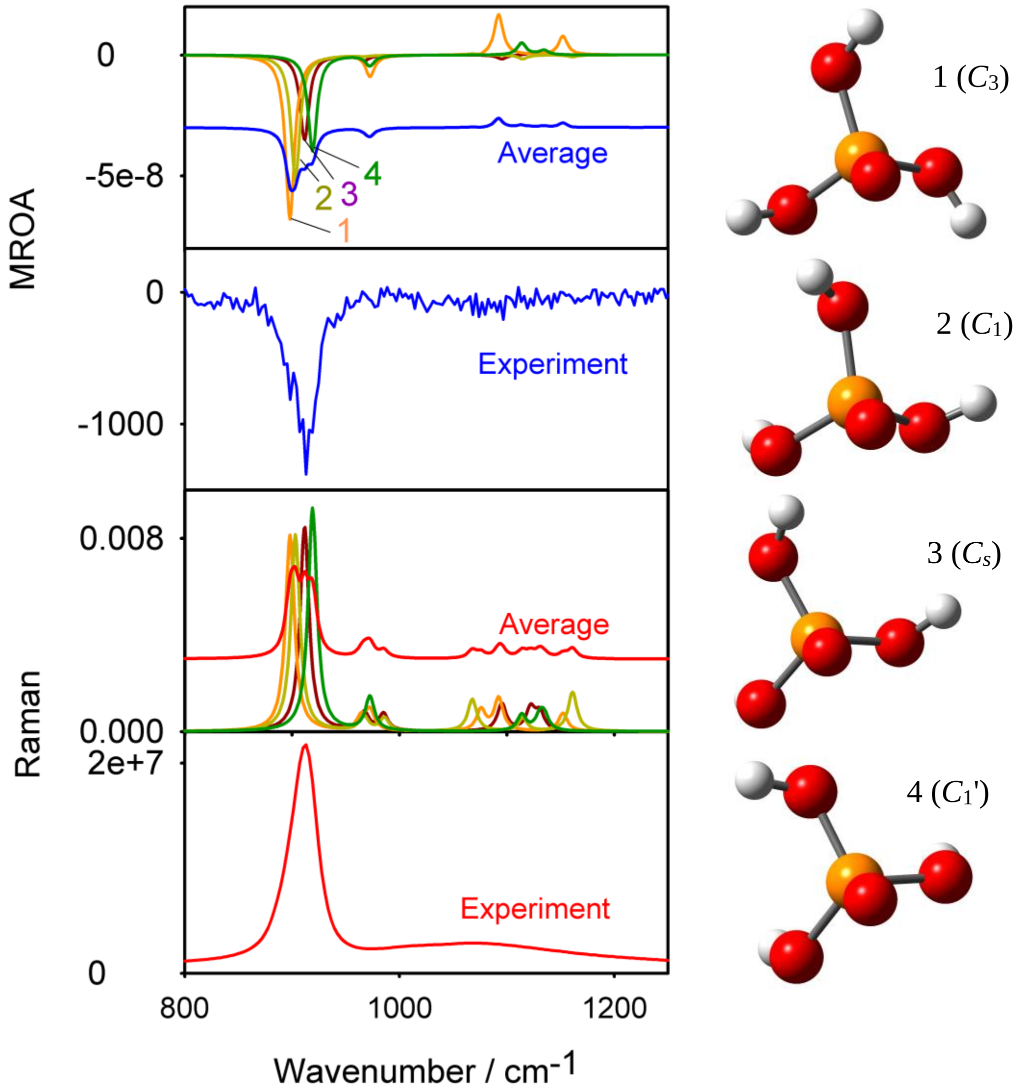


**Figure S3.** MROA a Raman spectra simulated (B3LYP/6-311++G**/PCM) for four conformers of phosphoric acid, and the experiment. Calculated frequencies are scaled by 0.9 for easier comparison.

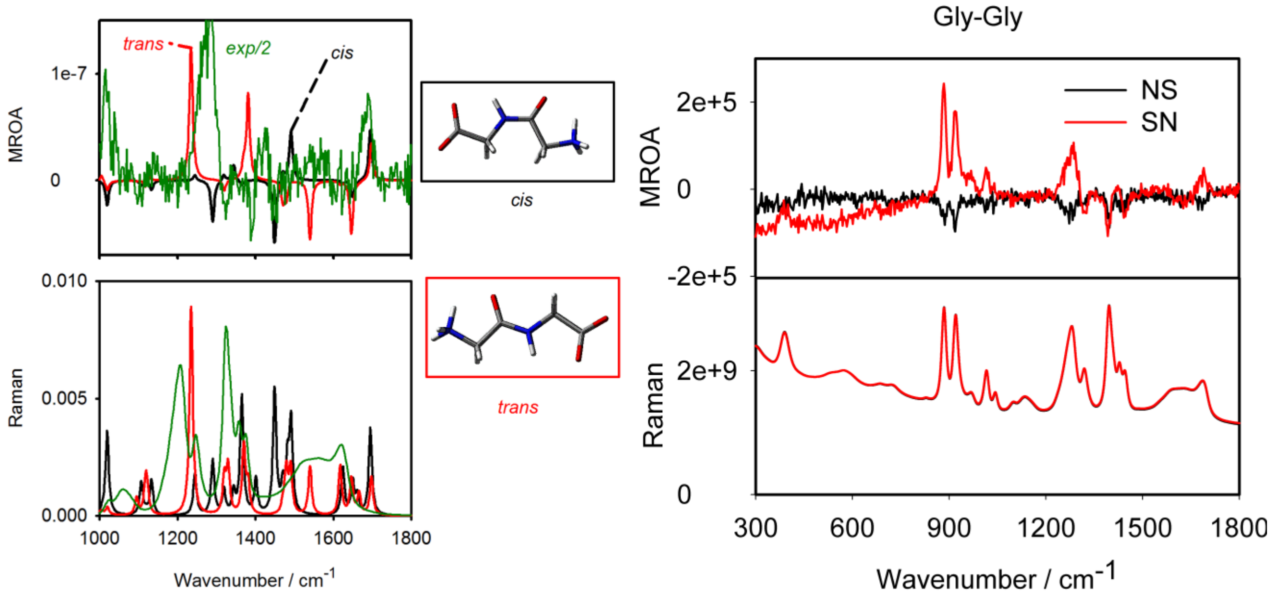


**Figure S4.** Left: MROA a Raman spectra simulated (B3LYP/6-311++G**/PCM) for cis and trans Gly-Gly conformers, and the experiment. Right: Experimental MROA and Raman spectra for two magnet orientations in a broader range, for 1 M water solution.

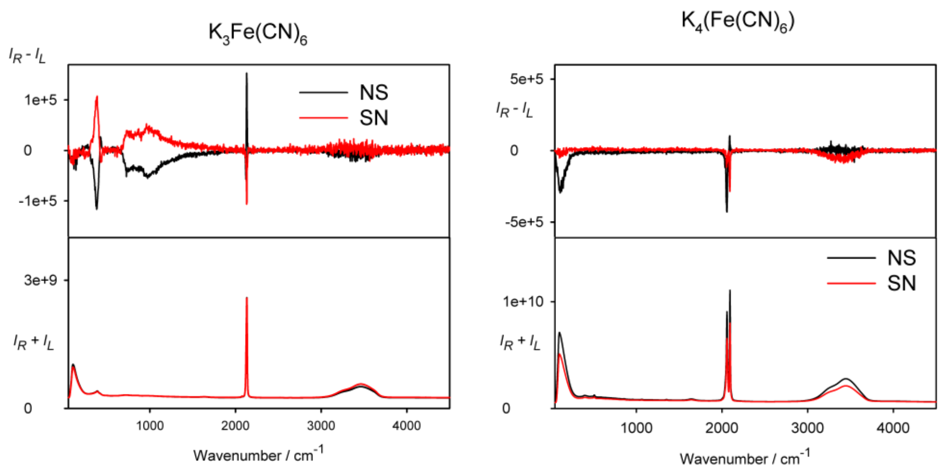


**Figure S5.** Raw Raman and MROA spectra of $K_3[Fe(CN)_6]$ and $K_4[Fe(CN)_6]$.

**DMSO:**

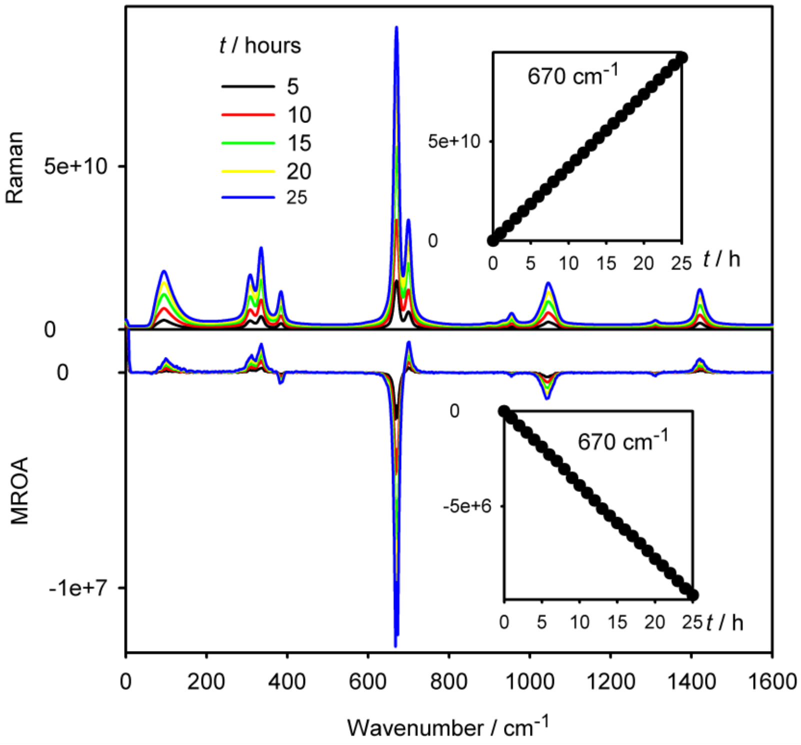


**$K_3[(CN)_6]$:**

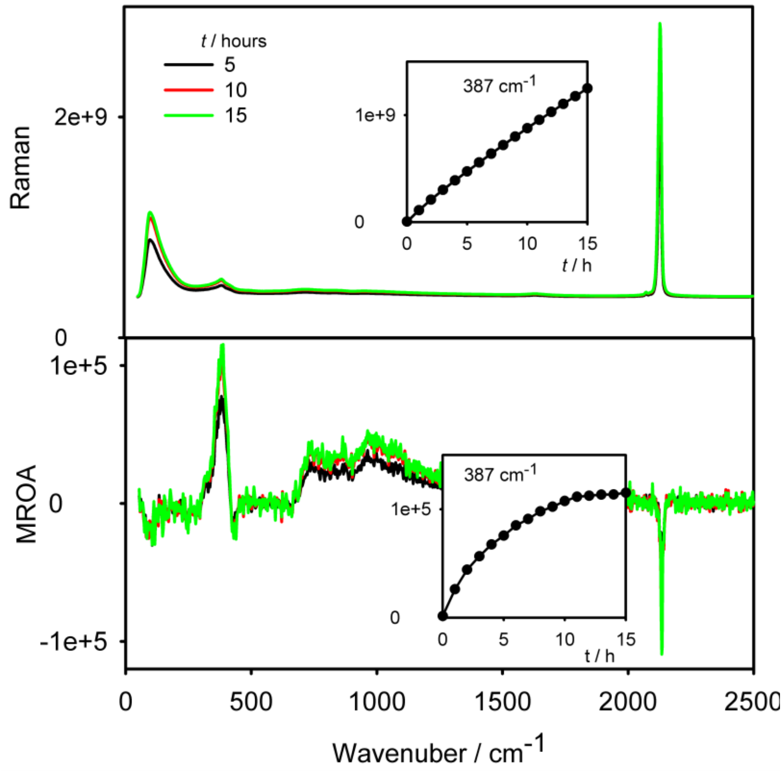


**Figure S6.** Time-dependence of Raman and MROA spectra during the accumulation, for DMSO and $K_3[(CN)_6]$, the insets show intensities in the indicated wavenumbers.

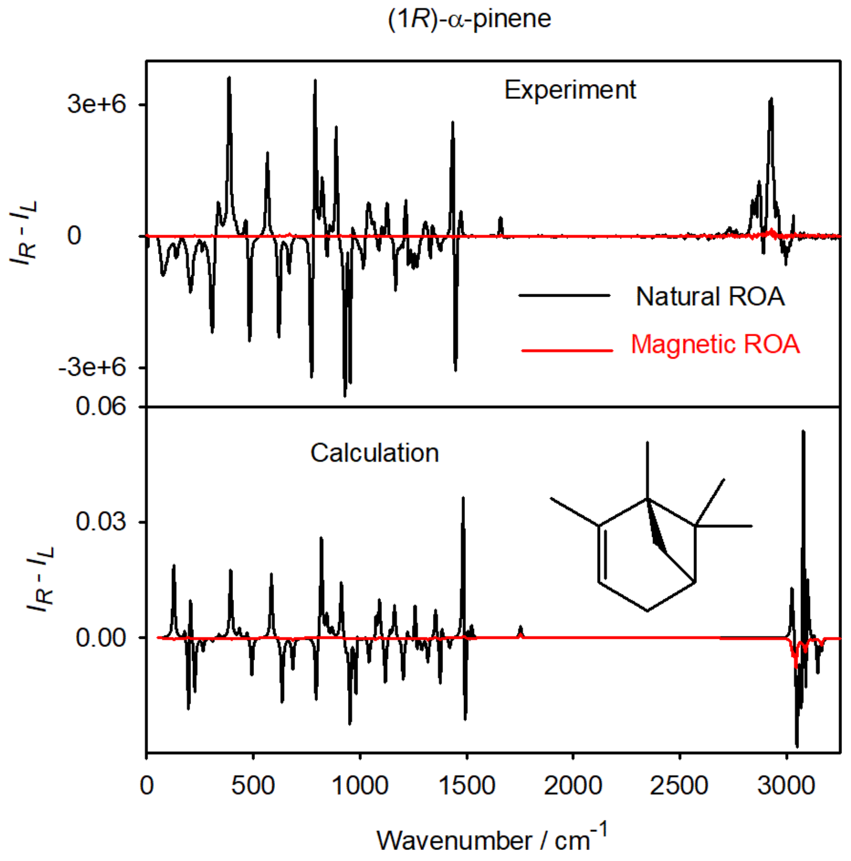


**Figure S7.** Calculated and experimental ROA and MROA spectra of α-pinene.

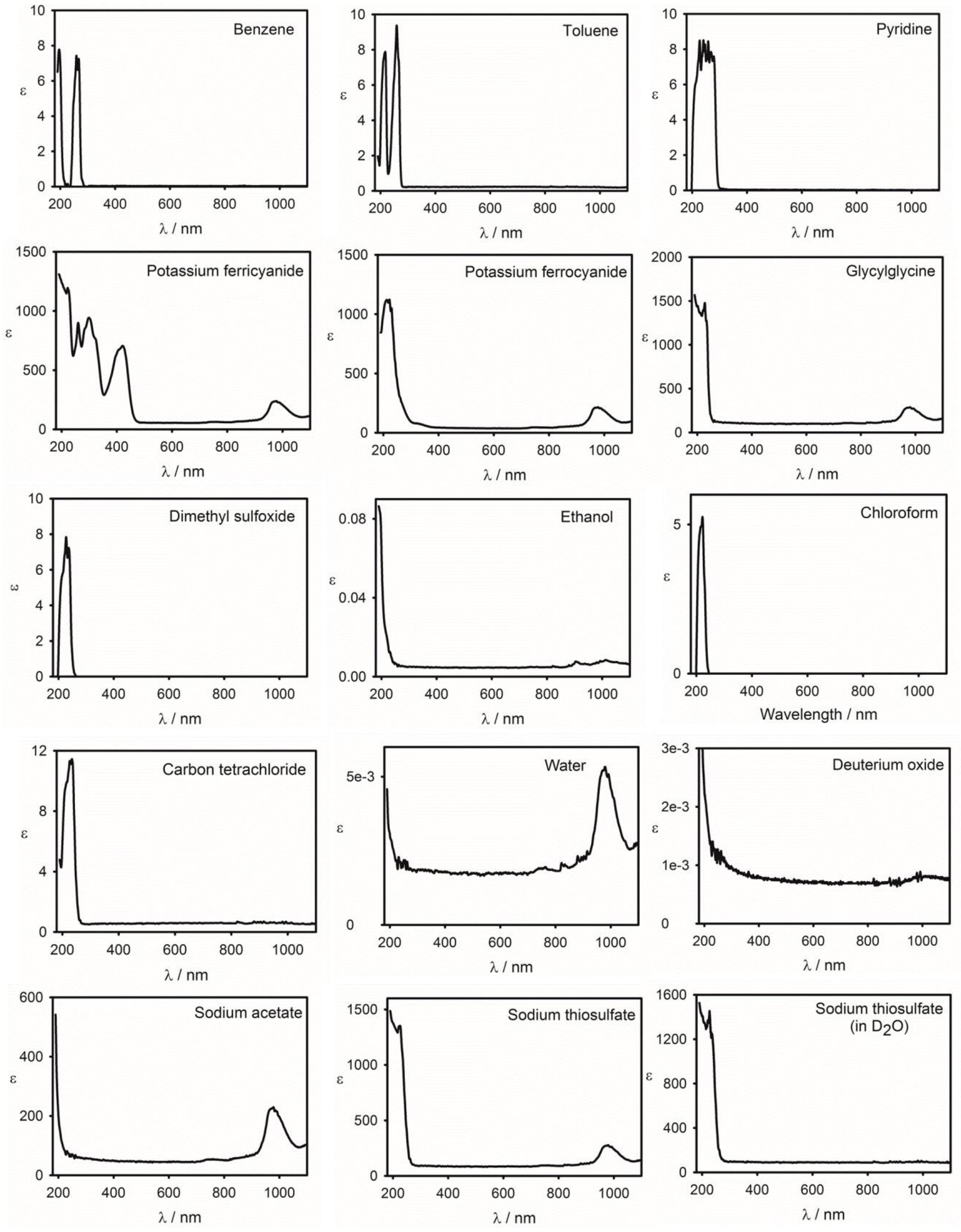


**Figure S8.** Experimental UV/vis absorption spectra of measured systems, in $L{\cdot}mol^{-1}{\cdot}cm^{-1}$.

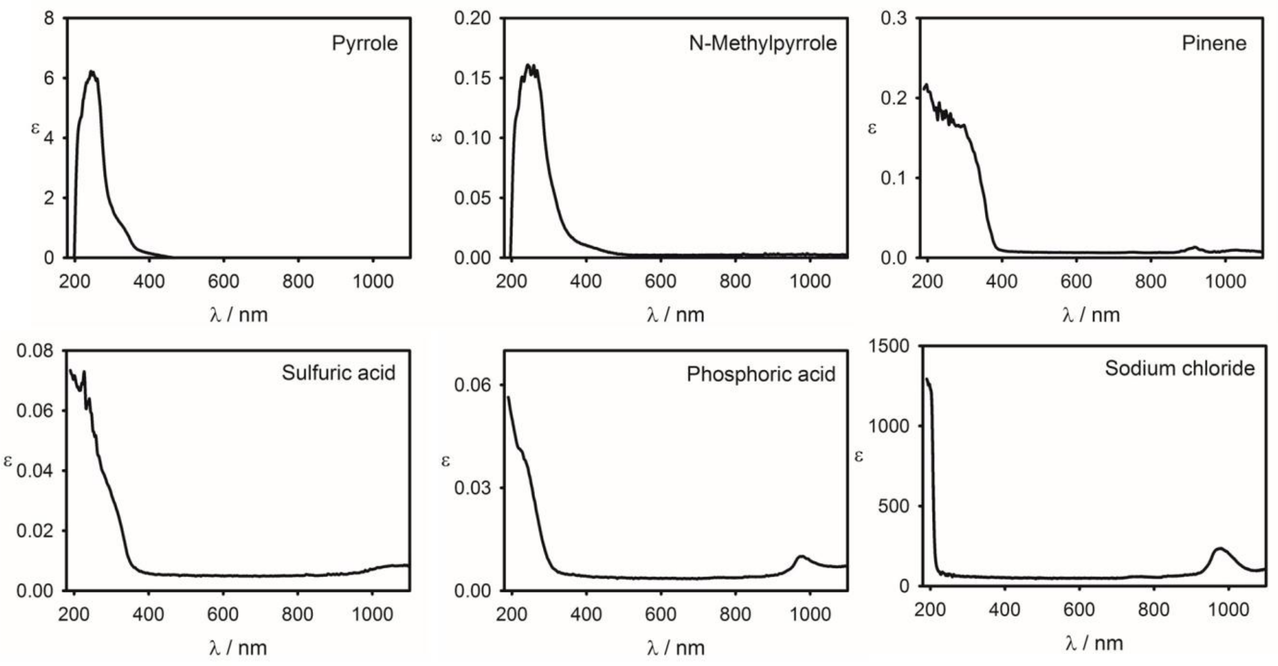


**Figure S8 (cont.).** Experimental UV/vis absorption spectra of measured systems.

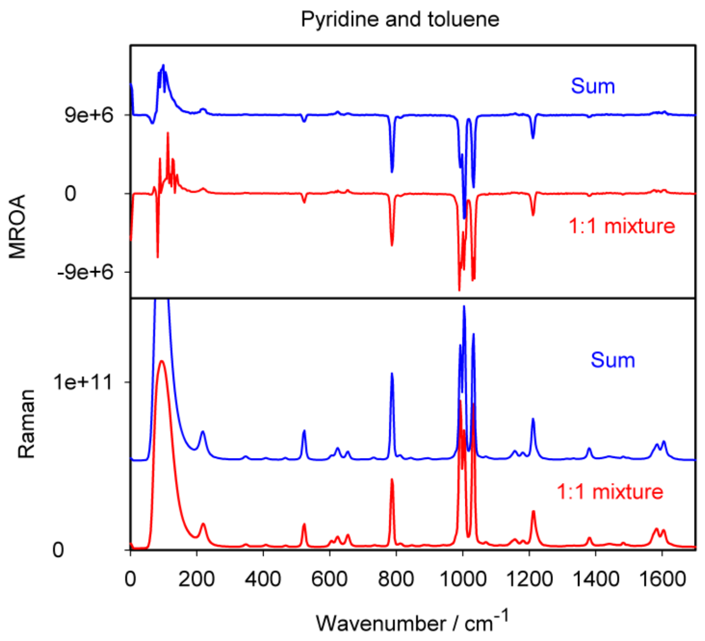


**Figure S9.** Experimental MROA and Raman spectra of pyridine and toluene, 1:1 mixture vs. plain sum of spectra of neat compounds.

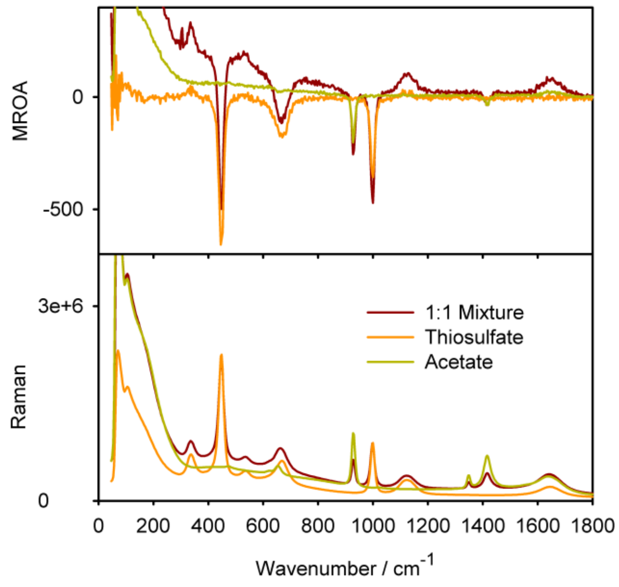


**Figure S10.** MROA and Raman spectra of 1:1 sodium thiosulfate and acetate mixture, vs. spectra of pure compounds. (Here MROA spectra are not compensated for two magnet orientations.)

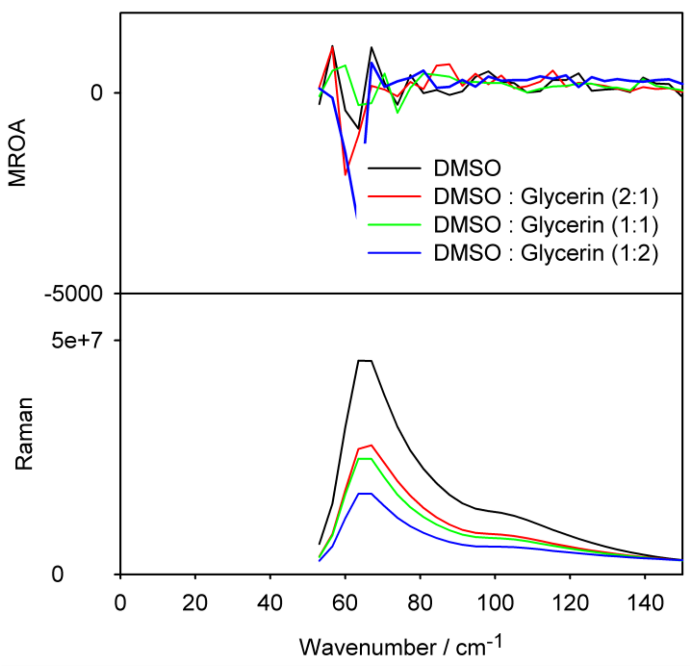


**Figure S11.** MROA and Raman spectra of various DMSO/glycerin mixtures, a low-frequency part.

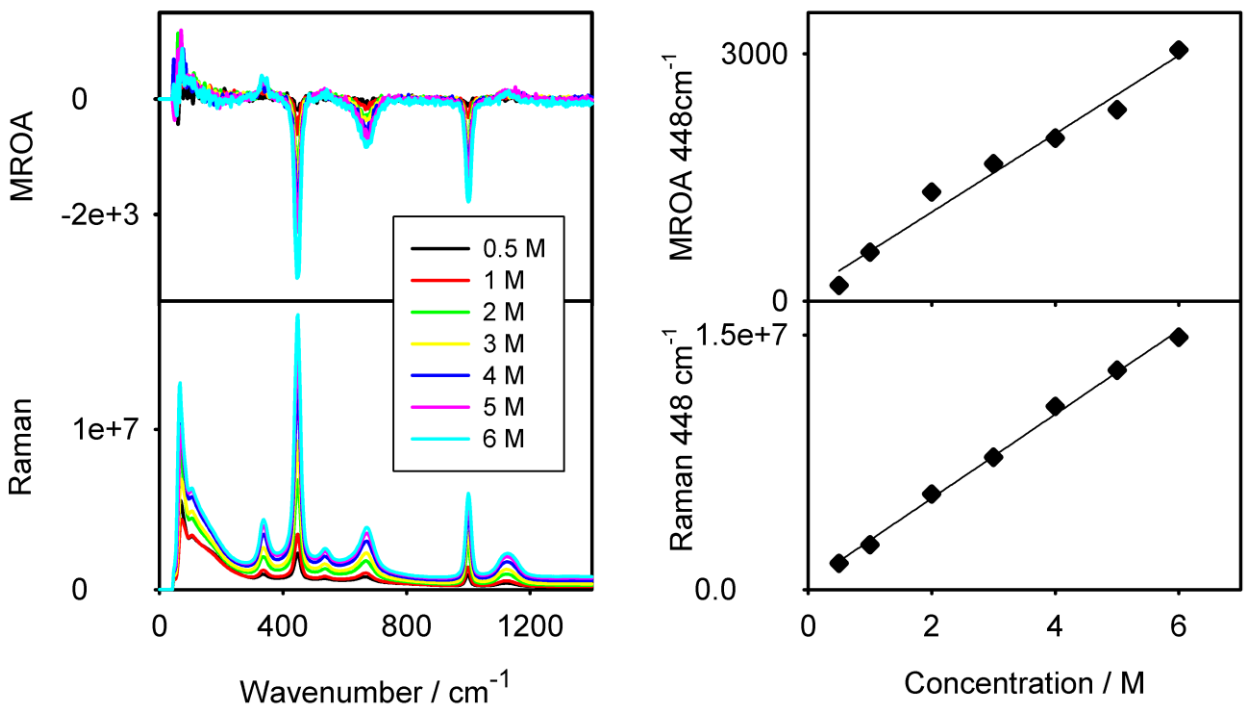


**Figure S12.** (Left:) concentration-dependence of MROA and Raman spectra of sodium thiosulfate, (right:) and of the 448 $cm^{-1}$ intensities.

## Spectra measurement

All chemicals were obtained from Sigma-Aldrich. Raman and ROA spectra were measured on a Zebr spectrometer[1] at 20°C, within 50 to 4550 $cm^{-1}$, using 532 nm excitation wavelength, the back-scattering geometry, and the scattered circular polarization (SCP) modulation scheme. The samples were held in a 10 mm quartz cell from Hellma Analytics (600 μL), in a magnetic compartment.[2] The laser power at the sample ranged from 60 to 250 mW and the data was collected for 7 to 22 hours for different samples for each magnet orientation. Idealized spectra for the "north-south" magnetic orientation ["(NS-SN)/2" for MROA and "(NS+SN)/2" for Raman] are presented in the main text. For optically active α-pinene, natural ROA spectrum was obtained simultaneously during MROA measurement, from the "(NS+SN)/2" sum of the ROA trace.

UV-Vis spectra (**Figure S8**) were measured on a WTW PhotoLab7600 spectrometer and expressed as the absorption index, $\varepsilon$ / $M^{-1}$ $cm^{-1}$.

For mixtures, either we did not observe other than additive effects, considering the accuracy of the measurement, e.g. in the pyridine and toluene (**Figure S9**), thiosulfate and acetate (**Figure S10**), and DMSO and glycerin (**Figure S11**) mixtures. $D_2O$ and NaCl $H_2O$ solutions did not brink results significantly different from $H_2O$. Except for $K_3[(CN)_6]$ we did not observe a significant time dependence of the spectra indicating sample decomposition; this could be conveniently tracked in the process of spectral accumulation (cf. **Figure S12**). Within the experimental error the spectra depended on the concentration linearly (**Figure S13**).

Specific measurement conditions were:

| Compound | Concentration / mol/L | Accumulation time / h | Laser power / mW |
|---|---|---|---|
| Benzene | neat | 21 | 60 |
| Toluene | neat | 20 | 100 |
| Pyridine | neat | 12 | 80 |
| α-pinene | neat | 12 | 80 |
| Glycylglycine | 1 | 22 | 250 |
| Potassium ferrocyanide | 0.8 | 7 | 250 |
| Potassium ferricyanide | 1 | 10 | 180 |
| Pyrrole | neat | 8 | 30 |
| N-methylpyrrole | neat | 3 | 50 |
| Dimethyl sulfoxide | neat | 24 | 60 |
| Carbon tetrachloride | neat | 1 | 80 |
| Ethanol | neat | 18 | 200 |

| | | | |
|---|---|---|---|
| Sulfuric acid | neat | 1 | 100 |
| Phosphoric acid | neat | 1.5 | 100 |
| Sodium acetate | 1 | 18 | 300 |
| Sodium thiosulfate ($H_2O$) | 1 | 10 | 200 |
| Sodium thiosulfate ($D_2O$) | 1 | 10 | 200 |
| Pyridine + Toluene (1:1) | neat | 22 | 100 |
| Sodium acetate + Sodium thiosulfate (1:1) | 1, 1 | 23 | 200 |
| Sodium thiosulfate | 0.5, 1, 2, 3, 4, 5, 6 | 1.5 each | 200 |
| Dimethyl sulfoxide : Glycerin 1:0, 2:1, 1:1, 1:2 | neat | 1.5 each | 60 |
| $H_2O$ | | 10 | 200 |
| $D_2O$ | | 11 | 200 |
| Sodium chloride ($H_2O$) | 1 | 5 | 200 |

## Theory of magnetic Raman optical activity

General formulae have been given in ref. [3]. We simplify them for a high-temperature case when we neglect orientation effects of the molecules in the magnetic field. Otherwise we consider a general case when the incident light with a wave vector **k** interacts with a molecule (*M*), and the scattered light with wave vector **k'** is detected (**Figure S14**). The wave vectors define the scattering angle $\xi$. Within the multipole approximation, the molecule is polarized by the incident light. Its electric intensity is **E**, and the intensity of the scattered light **E'** is related to it by a linear equation. In a complex representation,

$$E'_{\alpha} = Ke^{i(kr-\omega t)}a_{\alpha\beta}E_{\beta} \quad \text{(s1)}$$

where , $K = \dfrac{\omega^2 \mu_0}{4\pi r}$, $\omega$ is the frequency of the radiation, $\mu_0$ is vacuum permeability, $r$ is the distance from the molecule, $t$ is time, and **a** is a complex scattering tensor. In the simplest dipole approximation the tensor is equal to electric dipole - electric dipole polarizability, $\mathbf{a} = \boldsymbol{\alpha}$.

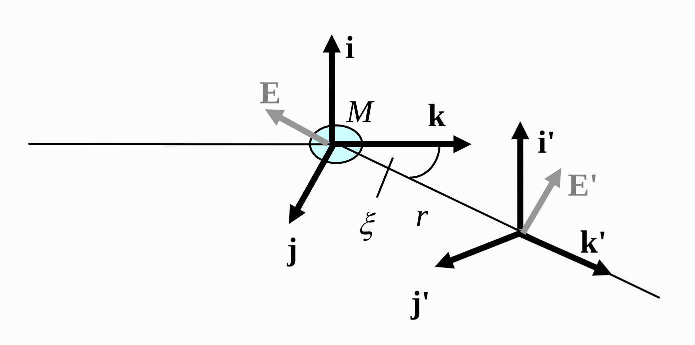


**Figure S13.** Raman scattering on a molecule.

To describe the MROA experiment, we need two components of the Stokes vector for the scattered radiation based on the incident Stokes vector (S0, S1, S2, S3),

$$
\begin{aligned}
S'_0 = \frac{1}{2}K^2\{ & (|a_{xx}|^2 + |a_{xy}|^2)S_0 + (|a_{xx}|^2 - |a_{xy}|^2)S_1 - 2\,\mathrm{Re}(a_{xx}a_{xy}^*)S_2 - 2\,\mathrm{Im}(a_{xx}a_{xy}^*)S_3 \\
& + [(|a_{yx}|^2 + |a_{yy}|^2)S_0 + (|a_{yx}|^2 - |a_{yy}|^2)S_1 - 2\,\mathrm{Re}(a_{yx}a_{yy}^*)S_2 - 2\,\mathrm{Im}(a_{yx}a_{xy}^*)S_3]\cos^2\xi \\
& + [(|a_{zx}|^2 + |a_{zy}|^2)S_0 + (|a_{zx}|^2 - |a_{zy}|^2)S_1 - 2\,\mathrm{Re}(a_{zx}a_{zy}^*)S_2 - 2\,\mathrm{Im}(a_{xz}a_{zy}^*)S_3]\sin^2\xi \\
& 2[\mathrm{Re}(a_{yx}a_{zx}^* + a_{yy}a_{zy}^*)S_0 + \mathrm{Re}(a_{yx}a_{zx}^* - a_{yy}a_{zy}^*)S_1 - \mathrm{Re}(a_{yx}a_{zy}^* + a_{zx}a_{yy}^*)S_1 \\
& - \mathrm{Im}(a_{yx}a_{zy}^* + a_{zx}a_{yy}^*)S_3]\sin^2\xi\}
\end{aligned}
, \qquad \text{(s2)}
$$

and

$$
\begin{aligned}
S'_3 = K^2\{ & [\mathrm{Im}(a_{xx}a_{yx}^* + a_{xy}a_{yy}^*)S_0 + \mathrm{Im}(a_{xx}a_{yx}^* - a_{xy}a_{yy}^*)S_1 \\
& - \mathrm{Im}(a_{xx}a_{yx}^* - a_{yx}a_{xy}^*)S_2 + \mathrm{Re}(a_{xx}a_{yy}^* - a_{xy}a_{zy}^*)S_3]\cos\xi \\
& - [\mathrm{Im}(a_{xx}a_{zx}^* + a_{xy}a_{zy}^*)S_0 + \mathrm{Im}(a_{xx}a_{zx}^* - a_{xy}a_{zy}^*)S_1 \\
& - \mathrm{Im}(a_{xx}a_{zy}^* - a_{zx}a_{xy}^*)S_2 + \mathrm{Im}(a_{xx}a_{zy}^* - a_{zx}a_{xy}^*)S_1]\sin\xi\}
\end{aligned}
. \qquad \text{(s3)}
$$

In particular, for the most common backscattering ($\xi$ = 180°), we get

$$
\begin{aligned}
S'_0 = \frac{1}{2}K^2\{ & (|a_{xx}|^2 + |a_{xy}|^2)S_0 + (|a_{xx}|^2 - |a_{xy}|^2)S_1 - 2\,\mathrm{Re}(a_{xx}a_{xy}^*)S_2 - 2\,\mathrm{Im}(a_{xx}a_{xy}^*)S_3 \\
& + (|a_{yx}|^2 + |a_{yy}|^2)S_0 + (|a_{yx}|^2 - |a_{yy}|^2)S_1 - 2\,\mathrm{Re}(a_{yx}a_{yy}^*)S_2 - 2\,\mathrm{Im}(a_{yx}a_{xy}^*)S_3]
\end{aligned}
, \qquad \text{(s2')}
$$

and

$$
\begin{aligned}
S'_3 = K^2[ & -\mathrm{Im}(a_{xx}a_{yx}^* + a_{xy}a_{yy}^*)S_0 - \mathrm{Im}(a_{xx}a_{yx}^* - a_{xy}a_{yy}^*)S_1 \\
& + \mathrm{Im}(a_{xx}a_{yx}^* - a_{yx}a_{xy}^*)S_2 - \mathrm{Re}(a_{xx}a_{yy}^* - a_{xy}a_{zy}^*)S_3]
\end{aligned}
\qquad \text{(s3')}
$$

To mimic the common scattered circular polarized (SCP) light ROA experiment, let us suppose that the incident light is polarized along the *x*-axis (vector **i** in **Figure S1**), while it propagates along the *z*-axis, coinciding with wave vector **k**. The incident electric intensity is

$$
\mathbf{E} = E_0(1\,0\,0)e^{i(kr-\omega t)} \qquad \text{(s4)}
$$

which gives us $S_0 = E_x E_x^* + E_y E_y^* = E_y^2$, $S_1 = S_0$, $S_2 = S_3 = 0$,

$$S'_0 = K^2 (|a_{xx}|^2 + |a_{yx}|^2] S_0, \tag{s2''}$$

and

$$S'_3 = -2K^2 \operatorname{Im}(a_{xx} a_{yx}^*) S_0 . \tag{s3''}$$

For isotropic samples, the tensor products can be averaged over all molecular orientations using $\langle a_{xx} a_{xx}^* \rangle = \frac{1}{15}(a_{\alpha\alpha} a_{\beta\beta}^* + 2a_{\alpha\beta} a_{\alpha\beta}^*)$, $\langle a_{xy} a_{xy}^* \rangle = \frac{1}{30}(3a_{\alpha\beta} a_{\alpha\beta}^* - a_{\alpha\alpha} a_{\beta\beta}^*)$. We use the Einstein summation convention throughout. For a case without a magnetic field, we thus get the usual result[4] for unpolarized Raman backscattering, $S'_3 = 0$ and

$$I_0 = I_R + I_L = S'_0 = \frac{K^2}{30}(a_{\alpha\alpha} a_{\beta\beta}^* + 7a_{\alpha\beta} a_{\alpha\beta}^*) S_0 \tag{s5}$$

where the total intensity $I_0$ is a sum of intensities of the right and left circularly polarized light.

The magnetic field of intensity $B$ is applied along the $z$-axis. We consider a first-order change so that the polarizability in the magnetic field becomes

$$\alpha_{\alpha\beta} = \alpha_{\alpha\beta}^0 + \beta_{\alpha\beta,\gamma} B_\gamma \tag{s6}$$

where $\beta_{\alpha\beta,\gamma}$ are the components of the derivatives of the polarizability with respect to the magnetic field. The field has a negligible effect on the total scattered intensity $S'_0$, but it gives rise to the $S'_3$ component also in the isotropic case. Inserting (s6) into (s3''), we get

$$S'_3 = -2K^2 \operatorname{Im}(a_{xx} a_{yx}^*) S_0 = -2K^2 \operatorname{Im}[\alpha_{xx}^0 \alpha_{yx}^{0*} + (\beta_{xx,z} \alpha_{yx}^{*0} + \alpha_{xx}^0 \beta_{yx,z}^*) B_z] S_0 + o(B_z^2) \tag{s7}$$

For isotropic samples, we can use the averages $\langle \alpha_{xx}^{0*} \alpha_{yx}^{0*} \rangle = 0$,
$\langle \beta_{xx,z} \alpha_{yx}^{0*} \rangle = \frac{1}{30} \varepsilon_{\alpha\gamma\delta} [(\beta_{\alpha\beta,\delta} + \beta_{\beta\alpha,\delta}) \alpha_{\gamma\beta}^{0*} + \beta_{\beta\beta,\alpha} \alpha_{\delta\gamma}^{0*}]$, and
$\langle \alpha_{xx}^0 \beta_{yx,z}^* \rangle = \frac{1}{30} \varepsilon_{\alpha\gamma\delta} [(\alpha_{\alpha\beta}^0 + \alpha_{\beta\alpha}^0) \beta_{\gamma\beta,\delta}^* + \alpha_{\beta\beta}^0 \beta_{\delta\gamma,\alpha}^*]$, giving

$$S'_3 = -\tfrac{1}{15} K^2 \operatorname{Im} \varepsilon_{\alpha\gamma\delta} [(\beta_{\alpha\beta,\delta} + \beta_{\beta\alpha,\delta}) \alpha_{\gamma\beta}^{0*} + (\alpha_{\alpha\beta}^0 + \alpha_{\beta\alpha}^0) \beta_{\gamma\beta,\delta}^* + \beta_{\beta\beta,\alpha} \alpha_{\delta\gamma}^{0*} + \alpha_{\beta\beta}^0 \beta_{\delta\gamma,\alpha}^*] S_0 B_z \tag{s8}$$

For symmetric polarizability, $\alpha_{\alpha\beta}^0 = \alpha_{\beta\alpha}^0$, $\beta_{\alpha\beta,\delta} = \beta_{\beta\alpha,\delta}$, this simplifies to

$$I_{ROA} = I_R - I_L = S'_3 = \tfrac{2}{15} K^2 \operatorname{Im} \varepsilon_{\alpha\gamma\delta} (\beta_{\alpha\beta,\gamma} \alpha_{\delta\beta}^{0*} - \alpha_{\alpha\beta}^0 \beta_{\gamma\beta,\delta}^*) S_0 B_z \tag{s9}$$

## Computations of the derivatives of the polarizability with respect to the magnetic field

The transition polarizability from state $i$ to state $f$ is defined as[5]

$$\alpha_{\alpha\beta} = \frac{1}{\hbar}\sum_{e\neq i}\left(\frac{\langle f|\mu_\alpha|e\rangle\langle e|\mu_\beta|i\rangle}{\omega_{ei}-\omega-i\Gamma_e}+\frac{\langle f|\mu_\beta|e\rangle\langle e|\mu_\alpha|i\rangle}{\omega_{ei}+\omega'+i\Gamma_e}\right) \tag{s10}$$

where $i$, $e$, and $f$ denote the initial, intermediate, and final states, respectively, $\mu_\alpha$ is an α-component of the electric dipole moment operator **μ**, $\omega$ is the excitation frequency, $\omega_{ei} = \omega_e - \omega_i$, $\omega_e$ and $\omega_i$ are angular frequencies corresponding respectively to the intermediate ("excited") and initial states, respectively, $\omega'$ is the scattered frequency, and $\hbar$ is the reduced Planck constant. Note that $\omega = \omega' + \omega_f - \omega_i$. The imaginary term $i\Gamma_e$ is proportional to spectral width and accounts for the finite life time of the state $e$.[6-7]

Applying the Born-Oppenheimer approximation, setting $\omega \sim \omega'$, $\Gamma_e = \Gamma$ for all states, and neglecting vibrational contributions and energy changes in the denominator, we get

$$\alpha_{\alpha\beta} = \frac{1}{\hbar}\sum_{e\neq 0}\left(\frac{\langle m|\mu_{0e\alpha}\mu_{e0\beta}|n\rangle}{\omega_{e0}-\omega-i\Gamma}+\frac{\langle m|\mu_{0e\beta}\mu_{e0\alpha}|n\rangle}{\omega_{e0}+\omega+i\Gamma}\right) \tag{s11}$$

where $n$ and $m$ are the initial and final vibrational states, the matrix element $\mu_{0e\alpha} = \langle 0|\mu_\alpha|e\rangle$ contains the ground and excited electronic wave functions, $\omega_{e0}$ is the difference of excited and ground electronic energies. At the magnetic field, we consider changes to the electronic structure so that

$$\alpha_{\alpha\beta}(B_\gamma) = \frac{1}{\hbar}\sum_{e\neq 0}\left(\frac{\langle m|\mu_{0e\alpha}(B_\gamma)\mu_{e0\beta}(B_\gamma)|n\rangle}{\omega_{e0}(B_\gamma)-\omega-i\Gamma}+\frac{\langle m|\mu_{0e\beta}(B_\gamma)\mu_{e0\alpha}(B_\gamma)|n\rangle}{\omega_{e0}(B_\gamma)+\omega+i\Gamma}\right) \tag{s12}$$

where $\mu_{0e\alpha}(B_\gamma) = \langle 0(B_\gamma)|\mu_\alpha|e(B_\gamma)\rangle$. This can be approximately[5] written as

$$\alpha_{\alpha\beta}(B_\gamma) \approx \langle m|\alpha_{E,\alpha\beta}(B_\gamma)|n\rangle \tag{s13}$$

where

$$\alpha_{E,\alpha\beta}(B_\gamma) = \frac{1}{\hbar}\sum_{e\neq 0}\left(\frac{\mu_{0e\alpha}(B_\gamma)\mu_{e0\beta}(B_\gamma)}{\omega_{e0}(B_\gamma)-\omega-i\Gamma}+\frac{\mu_{0e\beta}(B_\gamma)\mu_{e0\alpha}(B_\gamma)}{\omega_{e0}(B_\gamma)+\omega+i\Gamma}\right) \tag{s14}$$

is the electronic polarizability. For the harmonic approximation and a fundamental transition from the vibrational ground state to a single excited mode $i$,

$$\alpha_{\alpha\beta}(B_\gamma) = \frac{\partial \alpha_{E,\alpha\beta}(B_\gamma)}{\partial Q_i} \langle 1 | Q_i | 0 \rangle = \frac{\partial \alpha_{E,\alpha\beta}(B_\gamma)}{\partial R_\varepsilon} S_{\varepsilon i} \sqrt{\frac{\hbar}{2\omega_i}} Q_i \langle 1 | Q_i | 0 \rangle \quad \text{(s15)}$$

The derivatives were calculated by numerical differentiation,

$$\beta_{\alpha\beta,\gamma} = \frac{\partial \alpha_{\alpha\beta}}{\partial B_\gamma} = \frac{\alpha_{\alpha\beta}(B_\gamma) - \alpha_{\alpha\beta}(0)}{B_\gamma} \quad \text{(s16)}$$

where $\alpha_{\alpha\beta}(0)$ is the polarizability under zero magnetic field.

The Hamiltonian with the magnetic field is

$$H = H_0 - \mathbf{m} \cdot \mathbf{B} \quad \text{(s17)}$$

where $H_0$ is the unperturbed (Kohn-Sham, KS) Hamiltonian, **m** is the magnetic dipole moment operator, and **B** is the magnetic field. The electronic wave functions were chosen as

$$\Psi_e(\mathbf{B}) = \sum_J D_{eJ} \varphi_J \quad \text{(s18)}$$

where

$$\varphi_J = \sum_i c_{Ji} \Delta_i \quad \text{(s19)}$$

is the KS wave function for excited electronic state $J$, constructed from singly-excited Slater determinants $\Delta_j$, the index $j$ comprises excitations $a \to b$ from occupied to virtual orbitals. Note that within the KS theory, there is no recipe for an exact wave function; nevertheless, the single-excited representation (s19) reasonably well reproduces molecular properties.[8-9] The configuration interaction (CI) coefficients $c_{Ji}$ were obtained from density functional theory (DFT) computation, i.e., from the Gaussian[10] output. The $D_{eJ}$ coefficients and state energies $\varepsilon_e$ were obtained from the Schrödinger equation,

$$H\Psi_e(\mathbf{B}) = \varepsilon_e \Psi_e(\mathbf{B}) \quad \text{(s20)}$$

which leads to

$$\sum_J \langle \varphi_K | H | \varphi_J \rangle D_{eJ} = \varepsilon_e D_{eK} \tag{s20'}$$

i.e., to diagonalization of the Hamiltonian matrix. Note that the magnetic moment is a purely imaginary operator and that the resultant wave function is complex.

The dipole matrix elements needed in (s14) are thus

$$\mu_{0e\alpha}(B_\gamma) = \langle \Psi_0(B_\gamma) | \mu_\alpha | \Psi_e(B_\gamma) \rangle = \sum_{J,J'} D^*_{eJ} D_{eJ} \sum_{i,i'} c^*_{Ji} c_{J'i'} \langle \Delta_i | \mu_\alpha | \Delta_j \rangle \tag{s21}$$

Finally, the derivatives in (s15) were obtained by numerical differentiation with respect to nuclear coordinates $R_\varepsilon$,

$$\frac{\partial \alpha_{E,\alpha\beta}(B_\gamma)}{\partial R_\varepsilon} = \frac{\alpha_{E,\alpha\beta}(B_\gamma, R_\varepsilon + \Delta) - \alpha_{E,\alpha\beta}(B_\gamma, R_\varepsilon - \Delta)}{2\Delta} \tag{s22}$$

No numerical problems were observed with the numerical differentiation; in trial computations one- and two-step differentiation schemes provided virtually the same results. The default differentiation step was one tesla for the magnetic field and 0.05 Å for the coordinates**.**

## Origin dependence

The computed MROA intensities are in general dependent on the origin of coordinates. This is a general problem of properties dependent on the magnetic field if calculated with approximate wave functions, sometimes solvable by the introduction of the gauge-invariant atomic orbitals (GIAO, also called London or field-dependent AOs) or other orbital transformations.[11] In this work, we avoid the origin dependence of the results by placing the molecule in the center of mass.

We can nevertheless inspect the origin dependence more closely: In (s14), we inspect the dipole moment matrix elements. Since the perturbation potential is $V = -m_\gamma B_\gamma$ . From the perturbation theory we get the perturbed wave functions,

$$|0(B_\gamma)\rangle = |0^{(0)}\rangle - \sum_{e'\neq 0} \frac{\langle e'^{(0)} | m_\gamma B_\gamma | 0^{(0)} \rangle}{E_0^{(0)} - E_{e'}^{(0)}} | e'^{(0)} \rangle \tag{s23a}$$

$$\langle e(B_\gamma)| = \langle e^{(0)}| - \sum_{e'\neq e} \frac{\langle e^{(0)}|m_\gamma B_\gamma|e'^{(0)}\rangle}{E_e^{(0)} - E_{e'}^{(0)}} \langle e'^{(0)}| \tag{s23b}$$

and dipole moment (the $\gamma$-index indicates the derivative with respect to the magnetic field)

$$\mu_{e0\beta,\gamma} = \sum_{e'\neq 0} \frac{\langle 0^{(0)}|m_\gamma|e'^{(0)}\rangle}{E_0^{(0)} - E_{e'}^{(0)}} \langle e^{(0)}|\mu_\beta|e'^{(0)}\rangle - \sum_{e'\neq e} \frac{\langle e^{(0)}|m_\gamma|e'^{(0)}\rangle}{E_e^{(0)} - E_{e'}^{(0)}} \langle e'^{(0)}|\mu_\beta|0^{(0)}\rangle \tag{s24}$$

$$= \lim_{\eta\to 0}\left( \sum_{e'} \frac{m_{0e'\gamma}\mu_{ee'\beta}}{E_0^{(0)} - E_{e'}^{(0)} + \eta} - \sum_{e'} \frac{m_{ee'\gamma}}{E_e^{(0)} - E_{e'}^{(0)} + \eta} \mu_{e'0\beta} \right)$$

After an origin shift by **T**, the magnetic moment becomes

$$\mathbf{m}' = \sum_i \frac{q_i}{2m_i} \mathbf{r}'_i \times \mathbf{p}_i = \mathbf{m} + \mathbf{T} \times \sum_i \frac{q_i}{2m_i} \mathbf{p}_i \tag{s25}$$

so that

$$m'_{ee'\gamma} = m_{ee'\gamma} + \frac{1}{2} \varepsilon_{\gamma\delta\varepsilon} T_\delta \langle e^{(0)}| \sum_i \frac{q_i p_{i\varepsilon}}{m_i} |e'^{(0)}\rangle \tag{s26}$$

which, using the dipole-velocity transformation $i\langle e^{(0)}|\sum_i r_{i\varepsilon}|e'^{(0)}\rangle \frac{E_e^{(0)} - E_{e'}^{(0)}}{\hbar} = \langle e^{(0)}|\sum_i \frac{p_{i\varepsilon}}{m_i}|e'^{(0)}\rangle$ , becomes

$$m'_{ee'\gamma} = m_{ee'\gamma} + \frac{i}{2\hbar} \varepsilon_{\gamma\delta\varepsilon} T_\delta \mu_{ee'\varepsilon} (E_e^{(0)} - E_{e'}^{(0)}) \tag{s27}$$

Similarly, $m'_{0e'\gamma} = m_{0e'\gamma} + \frac{i}{2\hbar} \varepsilon_{\gamma\delta\varepsilon} T_\delta \mu_{0e'\varepsilon} (E_0^{(0)} - E_{e'}^{(0)})$, and

$$\mu'_{e0\beta,\gamma} = \mu_{e0\beta,\gamma} + \frac{i}{2\hbar}\varepsilon_{\gamma\delta\varepsilon}T_\delta \sum_{e'}(\mu_{0e'\varepsilon}\mu_{ee'\beta} - \mu_{ee'\varepsilon}\mu_{e'0\beta}) \quad \text{(s28)}$$

Using the resolution of identity, $\sum_{e'} |e'\rangle\langle e'| = 1$,

$$\mu'_{e0\beta,\gamma} = \mu_{e0\beta,\gamma} + \frac{i}{2\hbar}\varepsilon_{\gamma\delta\varepsilon}T_\delta \sum_{e'}\langle e|\mu_\varepsilon\mu_\beta - \mu_\varepsilon\mu_\beta|0\rangle = \mu_{e0\beta,\gamma} \quad \text{(s29)}$$

Therefore, in the limit of exact wave functions and a complete ensemble of molecular states, computed MROA becomes origin-independent.

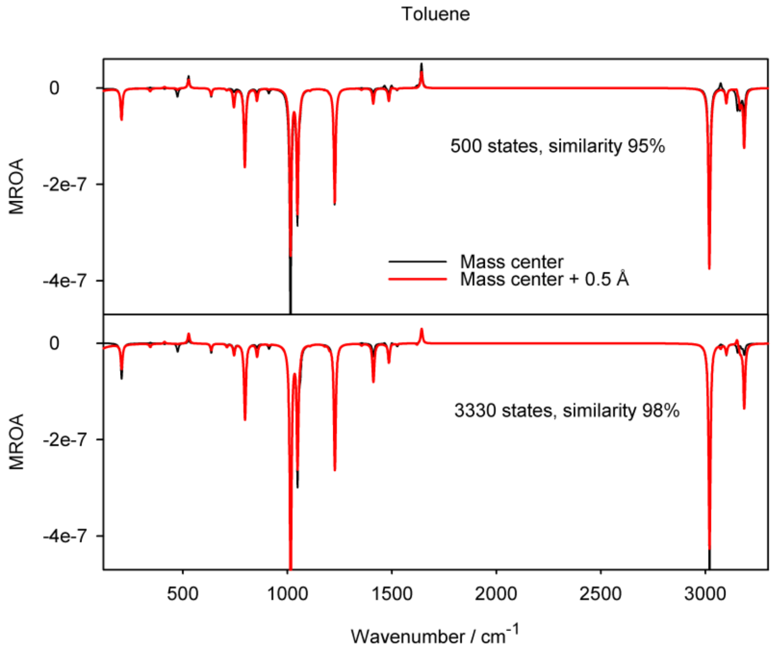


**Figure S14.** Toluene MROA spectra calculated for the origin in the center of mass and shifted by 0.5 Å, for 500 and 3330 electronic states included. The similarity between two spectra is defined as $s = \int S_1 S_2 d\omega / \sqrt{\int S_1^2 d\omega \int S_2^2 d\omega}$.

An example of the origin dependence of toluene MROA spectra calculated using the current implementation is given in **Figure S14.** For the small shift of 0.5 Å one can already see some changes in the band intensities, which are nevertheless acceptable for a semi-qualitative modeling needed for understanding the experimental signal and assignment of most vibrational bands. As predicted by the theory, the origin dependence is weaker when more excited electronic states are included in the calculations.

## Implementation

The Gaussian program was used for the quantum chemistry. MROA intensities were calculated at the harmonic approximation, using optimized geometries, polarizability derivatives ($\alpha^0$) and force fields calculated with the B3LYP[12] or CAM-B3LYP[13] functionals, in vacuum, or using the polarizable continuum model (PCM)[14] of solvent and the standard 6-311++G** basis set. The B3LYP and CAM-B3LYP results were quite similar, and only the former are reported. Excited electronic states and their CI coefficients were calculated at the same level, using time-dependent DFT. By our scripts, the dipole moment matrix elements, polarizability derivatives, and resultant Raman and MROA intensities were calculated. Smooth spectra $S(\omega)$ are plotted from the intensities $I_i$ of each transition using convolution with Lorentzian bands and a temperature correction[15]

$$S(\omega)=\sum_i I_i\left[1-\exp\left(-\frac{\omega_i}{kT}\right)\right]^{-1}\frac{2}{\pi\Delta}\left[4\left(\frac{\omega-\omega_i}{\Delta}\right)^2+1\right]^{-1} \quad \text{(s23)}$$

where $\omega$i is the vibrational frequency, k is the Boltzmann constant, T is temperature, and $\Delta$ = 10 cm$^{-1}$. As absolute intensities are not measured, calculated intensities were scaled to match the experimental ones. The same factor was used for Raman and MROA to preserve the CID ratio.

## References


[1] P. Michal, R. Čelechovský, M. Dudka, J. Kapitán, M. Vůjtek, M. Berešová, J. Šebestík, K. Thangavel, P. Bouř, "Vibrational Optical Activity of Intermolecular, Overtone, and Combination Bands: 2-Chloropropionitrile and α-Pinene" *J. Phys. Chem. B* **2019**, *123*, 2147.

[2] J. Šebestík, J. Kapitán, O. Pačes, P. Bouř, "Diamagnetic Raman Optical Activity of Chlorine, Bromine, and Iodine Gases" *Angew. Chem. Int. Ed.* **2016**, *55*, 3504.

[3] L. D. Barron, *Molecular Light Scattering and Optical Activity*, Cambridge University Press, Cambridge, UK, **2004**.

[4] L. Hecht, L. A. Nafie, "Theory of natural Raman optical activity" *Mol. Phys.* **1991**, *72*, 441.

[5] J. Šebestík, P. Bouř, "Quantum Chemical Computations of Raman Intensities: An Ambiguity of the Placzek Approximation" *Phys. Rev. A* **2025**, 062804.

[6] L. Nafie, "Theory of Raman scattering and Raman optical activity: near resonance theory and levels of approximation" *Theor. Chem. Account* **2008**, *119*, 39.

[7] L. Nafie, *Vibrational optical activity: Principles and applications*, Wiley, Chichester, **2011**.

[8] P. Štěpánek, P. Bouř, "Origin-Independent Sum Over States Simulations of Magnetic and Electronic Circular Dichroism Spectra via the Localized Orbital/Local Origin Method" *J. Comput. Chem.* **2015**, *36*, 723.

[9] P. Štěpánek, P. Bouř, "Computation of Magnetic Circular Dichroism by Sum Over States Summations" *J. Comput. Chem.* **2013**, *34*, 1531.

[10] M. J. Frisch, G. W. Trucks, H. B. Schlegel, G. E. Scuseria, M. A. Robb, J. R. Cheeseman, G. Scalmani, V. Barone, G. A. Petersson, H. Nakatsuji, X. Li, M. Caricato, A. V. Marenich, J. Bloino, B. G. Janesko, R. Gomperts, B. Mennucci, H. P. Hratchian, J. V. Ortiz, A. F. Izmaylov, J. L. Sonnenberg, D. Williams-Young, F. Ding, F. Lipparini, F. Egidi, J. Goings, B. Peng, A. Petrone, T. Henderson, D. Ranasinghe, V. G. Zakrzewski, J. Gao, N. Rega, G. Zheng, W. Liang, M. Hada, M. Ehara, K. Toyota, R. Fukuda, J. Hasegawa, M. Ishida, T. Nakajima, Y. Honda, O. Kitao, H. Nakai, T. Vreven, K. Throssell, J. A. Montgomery Jr., J. E. Peralta, F. Ogliaro, M. J. Bearpark, J. J. Heyd, E. N. Brothers, K. N. Kudin, V. N. Staroverov, T. A. Keith, R. Kobayashi, J. Normand, K. Raghavachari, A. P. Rendell, J. C. Burant, S. S. Iyengar, J. Tomasi, M. Cossi, J. M. Millam, M. Klene, C. Adamo, R. Cammi, J. W. Ochterski, R. L. Martin, K. Morokuma, O. Farkas, J. B. Foresman, D. J. Fox, Gaussian, Inc., Wallingford, CT, **2016**.

[11] K. Ruud, T. Helgaker, P. Bouř, "Gauge-origin independent density-functional theory calculations of vibrational Raman optical activity" *J. Phys. Chem. A* **2002**, *106*, 7448.

[12] A. D. Becke, "Density-functional thermochemistry. III. The role of exact exchange" *J. Chem. Phys.* **1993**, *98*, 5648.

[13] T. Yanai, D. Tew, N. C. Handy, "A new hybrid exchange-correlation functional using the Coulomb-attenuating method (CAM-B3LYP)" *Chem. Phys. Lett.* **2004**, *393*, 51.

[14] B. Mennucci, C. Cappelli, R. Cammi, J. Tomasi, "Modeling solvent effects on chiroptical properties" *Chirality* **2011**, *23*, 717.

[15] P. L. Polavarapu, *Vibrational spectra: principles and applications with emphasis on optical activity, Vol. 85*, Elsevier, Amsterdam, **1998**.
